# Saline and N-acetylcysteine-based strategies and other approaches to prevent the risk of CA-AKI: a meta-analysis

**DOI:** 10.3389/fmed.2025.1608626

**Published:** 2025-11-12

**Authors:** I-Chen Lin, Wen-Wen Tsai, Vin-Cent Wu, Heng-Chih Pan, Min-Hsiang Chuang, Jui-Yi Chen

**Affiliations:** 1 Department of Internal Medicine, Chi Mei Medical Center, Tainan, Taiwan; 2 Department of Neurology, Chi-Mei Medical Center, Tainan, Taiwan; 3 Department of Internal Medicine, National Taiwan University Hospital, Taipei, Taiwan; 4 NSARF (National Taiwan University Hospital Study Group of ARF) and TAIPAI (Taiwan Primary Aldosteronism Investigators), Taipei, Taiwan; 5 Division of Nephrology, Department of Internal Medicine, Keelung Chang Gung Memorial Hospital, Keelung, Taiwan; 6 Division of Nephrology, Department of Internal Medicine, Chi Mei Medical Center, Tainan, Taiwan; 7 Department of Health and Nutrition, Chia Nan University of Pharmacy and Science, Tainan, Taiwan

**Keywords:** network meta-analysis, contrast media, acute kidney injury, coronary angiography, combination strategies

## Abstract

**Background:**

While hydration is currently the most evidence-supported strategy for preventing contrast-associated acute kidney injury (CA-AKI) in patients undergoing cardiovascular angiography, the potential benefits of combining a saline and N-acetylcysteine (NAC) based strategy with additional pharmacologic interventions remain uncertain.

**Methods:**

We conducted a search for randomized controlled trials (RCTs) in PubMed, Embase, and the Cochrane library from the inception to 26th January 2024. RCTs involving adults undergoing cardiovascular angiography were analyzed, comparing the effects of saline and NAC-based strategies combined with additional agents compared to saline. The primary outcome was the risk of CA-AKI. The comparative effectiveness was visually represented through a network diagram and forest plot, with the treatments ranked by P-score in a league table.

**Results:**

We included 72 trials with 14,671 patients, 1,843 AKI events, comparing 12 different interventions based on hydration and NAC. The incidence of CA-AKI was 11.74% in the hydration with oral NAC group versus 15.49% in the hydration with saline alone group (odds ratio [OR] 0.78, 95% confidence interval [CI] 0.62–0.97). Compared to individuals with saline alone, the incidence of CA-AKI in the hydration with intravenous NAC group was 10.62% (OR 0.71, 95% CI 0.52–0.99); In hydration with oral NAC and statin group, the incidence of CA-AKI was 8.28% (OR 0.47, 95% CI 0.29–0.77).

**Conclusion:**

This network meta-analysis highlights that the combination of hydration with oral or intravenous NAC is more effective than hydration alone in preventing CA-AKI. Additionally, hydration with oral NAC and a statin significantly outperforms hydration with oral NAC alone in preventing CA-AKI.

**Systematic review registration:**

CRD42024502497.

## Introduction

Coronary angiography (CAG) or percutaneous intervention (PCI) is essential for diagnosing coronary artery disease (CAD) or treating acute coronary syndrome (ACS). However, both procedures rely on iodinated contrast media, which can potentially cause contrast-associated acute kidney injury (CA-AKI) (1), especially in patients with impaired kidney function (2). There is evidence that contrast media contribute to CA-AKI by reducing renal function through a combination of renal vasoconstriction, leading to hypoxia, and direct toxicity to tubular epithelial cells (3).

Previous studies have proposed various strategies for preventing CA-AKI, with hydration emerging as the most recommended approach in current guidelines (4–9) because sufficient hydration reduces urine viscosity after contrast administration (10) to decrease the risk of CA-AKI.

Despite this, reactive oxygen species (ROS) accumulate in chronic kidney disease (CKD), damaging cellular proteins and organelle membranes with highly reactive molecules like hydrogen peroxide and hydroxyl radicals (11). This damage indirectly affects microcirculation by producing vasoconstrictors such as endothelin and angiotensin II. N-acetylcysteine (NAC) helps counteract these effects by acting as a potent antioxidant (12, 13). However, current guidelines (4–9) tend not to recommend its use due to discordant and uncertain results observed in numerous randomized controlled trials (RCTs) (14–22) or meta-analyses (23, 24). The diversity of these results might be explained by different patient populations, variable dosing regimens, and/or concomitant infusions of balanced solutions.

In addition, statins exert a range of effects, including anti-inflammatory actions, vasodilation, and inhibition of pro-apoptotic processes (25–27). These effects contribute to improved endothelial function, increased nitric oxide availability to renal capillaries, and reduced inflammation. This is demonstrated in the JUPITER study (28), which showed decreased hs-C-reactive protein levels. Additionally, statins reduce the expression of endothelial angiotensin receptors and inhibit endothelin synthesis, potentially lowering the risk of CA-AKI (29, 30). Recent meta-analyses (26, 31, 32)have indicated that statins provide a protective effect against CA-AKI. Nevertheless, data on the combined preventive effects of statins remain limited. Previous studies (33, 34) have assessed the efficacy of various strategies in preventing CA-AKI and established rankings. However, these network meta-analyses primarily focused on single agents or limited pairwise comparisons, without systematically evaluating multi-component strategies such as saline-based hydration combined with NAC and/or statins. Moreover, prior analyses did not adequately explore the role of key effect modifiers (e.g., baseline CKD severity, contrast volume) that may influence treatment response. Therefore, our study extends existing evidence by conducting a comprehensive network meta-analysis (NMA) that integrates hydration, NAC, statins, and their combinations into a single network, allowing for a comparative ranking of preventive strategies while addressing important gaps left by previous meta-analyses.

## Materials and methods

### Identification and selection of studies

The systematic review followed the Preferred Reporting Items for Systematic Reviews and Meta-Analyses (PRISMA) guidelines (35). We conducted a search for RCTs in PubMed, Embase, and the Cochrane library without any date or language restrictions. The final search was conducted on January 26, 2024. Search terms combined free text and Medical Subject Heading (MeSH) terms. Two reviewers (ICL, WWT), independently screened citations using predefined selection criteria. The systematic review and protocol were registered with the PROSPERO registry CRD42024502497.

### Population

Studies were included the adults aged more than 18 years old undergoing contrast-enhanced intra-arterial procedures, such as CAG, percutaneous intervention (PCI), peripheral angiography (PA), and endovascular aneurysm repair (EVAR).

### Types of intervention and outcomes

We assessed studies comparing saline-based strategies combined with intravenous (IV) or oral form NAC and other supplementary interventions, including sodium bicarbonate (SB), febuxostat (FBX), statin, remote ischemic preconditioning (RIPC), or sodium bicarbonate plus pentoxifylline (PTX), to those using only saline-based hydration in reducing the risk of CA-AKI.

We restricted our search to studies published in English due to feasibility of translation and resource limitations. We excluded studies enrolling patients younger than 18 years, those using intravenous contrast media, and non-RCT designs (e.g., cohort studies, case series, or case reports). Trials comparing different doses of the same intervention were also excluded. In addition, we excluded studies evaluating interventions with limited clinical applicability for CA-AKI prevention, such as theophylline or phentolamine, since these agents can independently increase GFR and potentially bias creatinine-based outcomes.

Various definitions of CA-AKI were considered in this review. Previously, CA-AKI was identified by an increase in serum creatinine of ≥0.5 mg/dL or a 25% rise from baseline within 2–5 days after contrast exposure (36). More recent guidelines define it as an increase of ≥0.3 mg/dL or a serum creatinine increase of ≥1.5–1.9 times baseline within 3 days of contrast medium administration, provided no alternative causes are evident (4). Definitions of CA-AKI mentioned above are widely used worldwide. Therefore, we accepted the original trial-specific definitions without re-defining outcomes, and extracted event numbers as reported.

### Data extraction and quality assessment

For each included study, data were recorded regarding the types of cardiovascular procedures (ex. CAG, PCI, PA, and EVAR), sample size, contrast type classified as high, iso, low osmolarity, types and dose of each intervention. Additionally, co-intervention with basic strategies, the number and mean age of each group, gender, contrast volume, and baseline kidney function were also recorded.

Trials were considered eligible if they reported intervention and comparator groups, provided a clear CA-AKI definition, and reported the number of CA-AKI events. Studies were included even when baseline renal function data were not available, provided that randomization appeared adequate and intervention/comparator groups were otherwise comparable. We documented whether baseline renal function information was reported, but did not exclude studies solely on this basis.

Furthermore, we statistically analyzed the proportions based on the year of publication of the included articles, geographical region (continent), study site (single-center, multicenter, or unknown), sample size, type of procedure with intra-arterial contrast, contrast type according to osmolarity, co-intervention described as basic treatment, mean serum creatinine levels.

### The geometry of the network

A network diagram was generated using the R tool to visually depict the trial’s size and the number of pairwise comparisons between interventions. The size of each intervention node corresponds to the number of patients included in the network, while the thickness of the interconnecting lines reflects the number of pairwise comparisons between any two interventions.

### Risk of bias

The risk of bias in the studies was assessed using the Risk of Bias tool 2.0 (RoB2) from Cochrane for randomized clinical trials. RoB2 assessments were conducted independently by two reviewers (ICL, WWT). In cases where there was disagreement between the two evaluators regarding the assessed risk of bias, a third reviewer facilitated consensus (JYC). The evaluators examined various domains including the randomization process, deviations from intended interventions, missing outcome data, measurement of outcomes, and selection of reported results. Subsequently, the studies were categorized into low, some concerns, or high risk of bias based on these assessments.

### Statistical analysis

We used the “meta” statistical package in R, version 4.3.1, provided by the R Foundation for Statistical Computing (37). We conducted standard meta-analyses by using a frequentist random-effects model to parameters reflecting the (pooled) relative treatment effect of each intervention compared with the reference treatment. We employing different control groups with hydration alone and hydration combined with oral NAC for the risk of CA-AKI. Outcomes from dichotomous data were reported as odds ratios (OR) with 95% confidence intervals (CI). Additionally, we synthesized evidence from the entire network using the netmeta command in R, integrating both direct and indirect estimates into a single summary effect in our random-effects NMA.

We assessed the probability of treatment ranking using P-scores and examined consistency within the network through the net-splitting method. Publication bias was evaluated by the comparison-adjusted funnel plot. Additionally, we conducted pre-specified subgroup analyses, stratified by various potential risk factors, such as CKD, low/iso osmolar contrast, volume exceeding 120 mL, and the studies conducted after 2010, to explore the risk of CA-AKI among different subgroups.

The plausibility of the transitivity assumption was assessed by comparing the distributions of potential effect modifiers across studies grouped by intervention. Two independent investigators (ICL and WWT) visually examined the distributions of potential effect modifiers across the interventions aimed at preventing CA-AKI and reached a consensus on whether substantial dissimilarities existed that could threaten the transitivity assumption. The potential effect modifiers showing dissimilarities were then evaluated using network meta-regression and sensitivity analyses to determine their influence.

Consistency across the entire network was evaluated by analyzing heterogeneity both within and between groups. An alternative estimation for the between-group heterogeneity was employed using the design-by-treatment interaction model. Furthermore, node splitting was applied to differentiate indirect evidence from direct evidence.

### Grading of evidence

We evaluated the quality of the evidence using the grading of recommendations assessment, development and evaluation (GRADE) framework, which involves assessing factors such as risk of bias, imprecision, inconsistency, indirectness, publication bias, intransitivity, and incoherence. Following GRADE guidelines, we interpreted and presented the NMA findings using a minimally contextualized framework to rate the effects (38–42). To assess publication bias, we reviewed trial registries for completed studies that did not have corresponding publications or reported results, again following the GRADE approach (41). To address local incoherence and obtain indirect estimates, we applied node-splitting models in line with GRADE recommendations.

## Results

### Study selection

**Figure 1:**
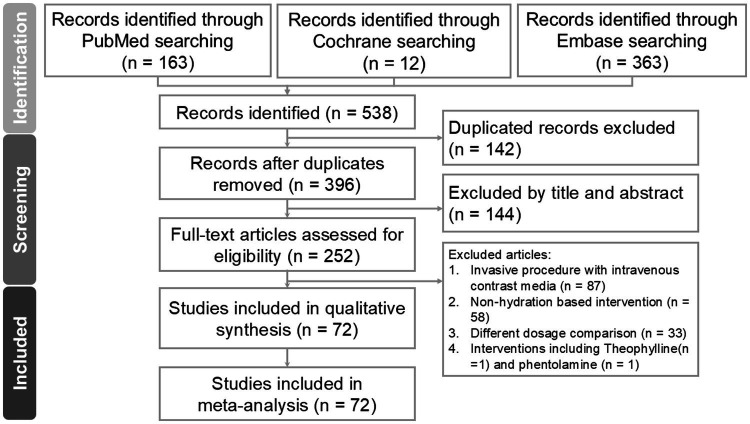
Identification and selection of studies for network meta-analysis.

A total of 538 study titles were identified in the initial literature search, out of which 72 met the inclusion criteria. The flowchart illustrating the search process is provided in Figure 1. After removing duplicates, 142 studies were excluded. Additionally, 144 studies were excluded based on their titles and abstracts not meeting our inclusion criteria. Subsequently, a total of 252 studies underwent full review. Reasons for exclusions included invasive procedures involving intravenous contrast injection (*n* = 87), interventions not based on hydration (*n* = 58), comparisons involving different dosages (*n* = 33), and inventions including theophylline(*n* = 1) and phentolamine (*n* = 1).

### Study characteristics

Our analysis included 74 RCTs with 14,671 patients and 1,843 AKI events, of which 62.07% (*n* = 9,405) were males. Due to some articles not providing individual counts of males and females, there is a discrepancy in the total count. Most RCTs were conducted in Asia (*n* = 31; 43.06%) and Europe (*n* = 21; 29.17%). Thirty-nine studies (54.17%) were single-center studies, while five (6.94%) were multicenter studies. CAG or/and PCI accounted for 63 (87.50%) of the invasive procedures with intra-arterial contrast. Low osmolar contrast media were used in 44 studies (61.11%), iso-osmolar agents in 16 studies (22.22%), and high-osmolar media in one study (1.39%). In addition, seven trials (9.72%) permitted physician discretion in the selection of contrast media, while a further 4 (5.56%) did not specify the contrast medium utilized. Most studies (27.78%) involved individuals with mean serum creatinine of 1.01–1.50 mg/dL. However, twenty-two studies (31.94%) were considered incomplete information for baseline kidney function (Table 1). Additional individual study characteristics are provided in Supplementary Table S1.

**Table 1:** Network characteristics.

Study characteristic	No. (%) of randomized clinical trials (*N* = 72)
Year of publication	
2001–2005	20 (27.78)
2006–2010	19 (26.39)
2011–2015	19 (26.39)
2016–2020	12 (16.67)
2021–2024	2 (2.78)
Continent	
Europe	21 (29.17)
North America	14 (16.44)
South America	2 (2.78)
Asia	31 (43.06)
Africa	2 (2.78)
Oceania	2 (2.78)
Site	
Single-center	39 (54.17)
Multicenter	5 (6.94)
Unknown	28 (38.89)
Sample size (no. of participants)	
0–50	13 (18.06)
51–100	18 (25.00)
101–150	12 (16.67)
151–200	7 (9.72)
>200	22 (30.56)
Procedure with intra-arterial contrast exposure	
CAG or/and PCI	63 (87.50)
PTCA	3 (4.17)
EVAR	1 (1.39)
Multiple^†^	5 (6.94)
Contrast type	
High osmolarity	1 (1.39)
Iso osmolarity	16 (22.22)
Low osmolarity	44 (61.11)
Mixed^*^	7 (9.72)
Unknown	4 (5.56)
Mean serum creatinine (mg/dl)	
≦1.00	8 (11.11)
1.01–1.50	20 (27.78)
1.51–2.00	14 (19.44)
≧2.01	7 (9.72)
Incomplete data^‡^	22 (31.94)
Total Number of Patients in Network	14,671
Total Number of CA-AKI in the network	1,843
Sex category^§^	
Male	9,405 (62.07)
Female	5,566 (37.93)

^*^Different types of contrast were chosen by the clinician. ^†^Procedures included at least two different types. ^‡^Some articles did not provide mean serum creatinine data, or only provided ClCr, eGFR, or median serum creatinine, which were excluded from the calculation. ^§^Some articles lacked complete separate numbers of males and females, which were excluded from the calculation. CA-AKI, Contrast-associated acute kidney injury; CAG, Coronary angiography; ClCr, Creatinine Clearance by Cockcroft-Gault Equation; eGFR, Estimated glomerular filtration rate; EVAR, Endovascular aneurysm repair; PCI, Percutaneous intervention; PTCA, Percutaneous transluminal coronary angioplast.

### Network structure and geometry

**Figure 2:**
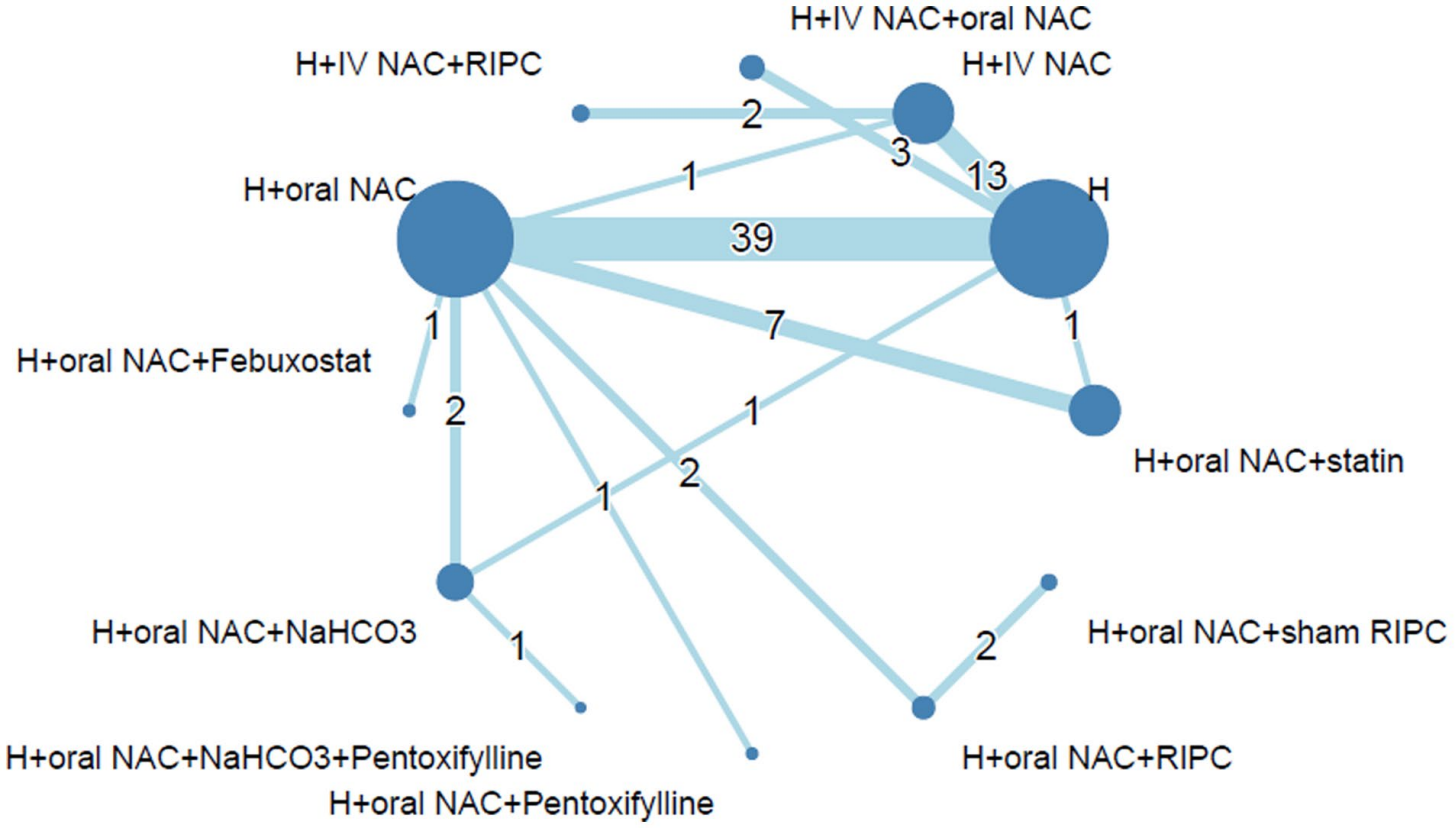
Network diagram of each intervention of prevention for CA-AKI. The size of each intervention node scales with the number of patients included in the trials, while the thickness of interconnecting lines corresponds to the number of pairwise comparisons between any two interventions. Abbreviations: CA-AKI, Contrast-associated acute kidney injury; H, Hydration; IV, Intravenous; NAC, N-acetylcysteine; RIPC, Remote ischemic preconditioning.

The relationship and comparisons between included studies are demonstrated in the network diagram (Figure 2). Fourteen interventions are included in this network. Data from 14,671 patients recruited to 72 trials investigating 12 interventions were included in our analysis. 170 pairwise comparisons were included, out of which 22 comparisons were excluded due to unfulfilled inclusion criteria. Detailed information is provided in Supplementary Table S1. The interventions most investigated were intravenous saline based hydration (H, *n* = 5,635), hydration plus oral N-acetylcysteine (H + oral NAC, *n* = 5,419), hydration plus intravenous N-acetylcysteine (H + IV NAC, *n* = 1,469), and hydration plus oral N-acetylcysteine and statin (H + oral NAC + statin, *n* = 954). The characteristics of individual interventions are outlined in Supplementary Table S2.

### Risk of bias

The risk of bias was assessed by two authors (VCW, HCP). A summary for individual studies is provided in Supplementary Figure S1. Most of the studies demonstrated “some concerns” to “low risk of bias.” Some of the included studies showed “some concerns” in Domain 2 (bias due to deviation from intended intervention) due to the lack of a control group or a complete description of co-intervention. Three studies (43–45) were classified as high risk of bias in the overall domain based on multiple domains classified as some concerns. As the outcome measure (CA-AKI) depends on laboratory results, it seems reasonable to assume that the risk of bias attributed to blinding of outcome assessment domain was low by default. The funnel plot for the assessment of publication bias of the included studies is shown in Supplementary Figure S2.

### Comparison of each intervention in preventing the risk of CA-AKI

**Figure 3:**
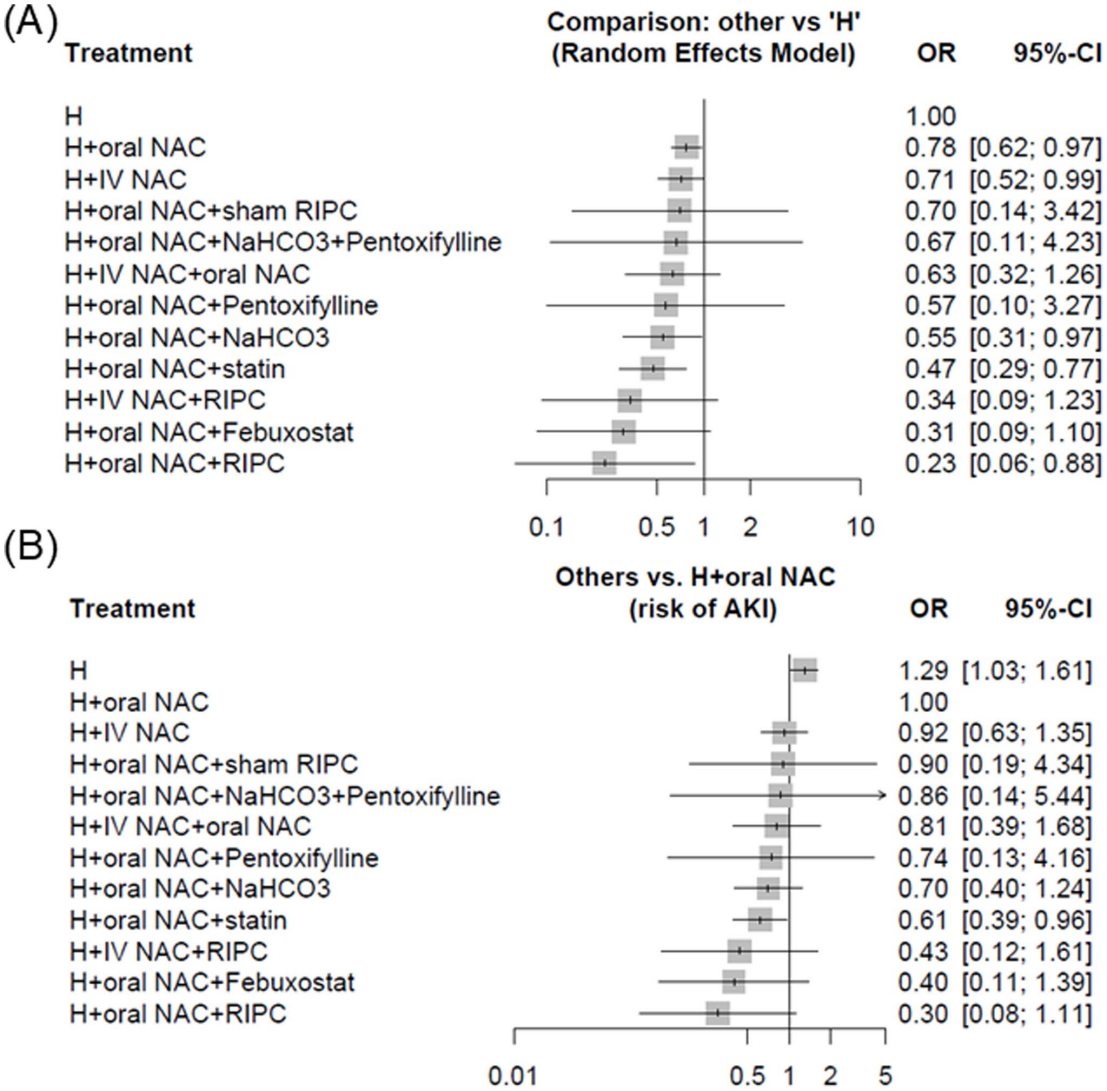
Forest plots demonstrating strategies for the prevention of CA-AKI among patients with cardiovascular angiography, employing different control groups **(A)** hydration alone and **(B)** hydration combined with oral N-acetylcysteine. Abbreviations: CA-AKI, Contrast-associated acute kidney injury; CI, Confidence interval; H, Hydration; IV, Intravenous; NAC, N-acetylcysteine; OR, Odds ratio; RIPC, Remote ischemic preconditioning.

Compared to hydration (H) with saline alone (the incidence of CA-AKI: 15.49%, 873 out of 5,635 patients), the combination of NAC, whether oral or intravenous route, demonstrated a benefit in preventing CA-AKI (H + oral NAC, 636 out of 5,419 patients, incidence 11.74%, OR: 0.78; 95% CI, 0.62–0.97, p score = 0.29; low-quality evidence; H + IV NAC, 156 out of 1,469 patients, incidence 10.62%, OR, 0.71; 95% CI, 0.52–0.99, p score = 0.36; high-quality evidence) (Figure 3A). Additionally, combining hydration with oral NAC, plus NaHCO3 (OR, 0.55; 95% CI, 0.31–0.97); plus statin (OR, 0.47; 95% CI, 0.29–0.77), indicating a significant benefit in preventing CA-AKI compared to hydration with saline alone. Besides, using hydration with oral NAC as the control, we observed a significantly greater effect in preventing CA-AKI with the combination of hydration, oral NAC, and statin (79 out of 954 patients, incidence 8.28%, OR: 0.61, 95% CI 0.39–0.96, *p* score = 0.63; very low-quality evidence) compared to hydration with oral NAC alone (Figure 3B). The results of both pairwise and network meta-analyses for risk of CA-AKI are provided in Table 2. The P score is utilized to determine the most effective prevention method for CA-AKI. Supplementary Table S3 presents a league table that summarizes the effect estimates and ranks the interventions according to their effectiveness in preventing CA-AKI.

**Table 2:** Network and pairwise meta-analyses for prevention of CA-AKI.

H + oNAC+RIPC	–	–	–	–	–	–	–	**0.33 (0.14–0.79)**	–	0.30 (0.08–1.11)	–
0.76 (0.12–4.64)	H + oNAC+FBX	–	–	–	–	–	–	–	–	0.40 (0.11–1.39)	–
0.69 (0.11–4.40)	0.91 (0.15–5.58)	H + iNAC+RIPC	–	–	–	–	–	–	0.47 (0.14–1.65)	–	–
0.49 (0.12–1.96)	0.65 (0.17–2.45)	0.71 (0.18–2.82)	H + oNAC+ST	–	–	–	–	–	–	**0.52 (0.33–0.84)**	1.76 (0.44–7.00)
0.43 (0.10–1.77)	0.56 (0.14–2.23)	0.62 (0.15–2.54)	0.87 (0.42–1.78)	H + oNAC+SB	–	–	0.82 (0.14–4.72)	–	–	0.60 (0.29–1.20)	0.74 (0.29–1.87)
0.41 (0.05–3.58)	0.54 (0.06–4.57)	0.59 (0.07–5.17)	0.83 (0.14–4.98)	0.96 (0.15–5.93)	H + oNAC+PTX	–	–	–	–	0.74 (0.13–4.16)	–
0.37 (0.08–1.65)	0.49 (0.11–2.08)	0.53 (0.12–2.32)	0.75 (0.32–1.76)	0.87 (0.35–2.14)	0.90 (0.14–5.92)	H + iNAC+oNAC	–	–	–	–	0.63 (0.32–1.26)
0.35 (0.04–3.34)	0.46 (0.05–4.27)	0.50 (0.05–4.80)	0.71 (0.11–4.73)	0.82 (0.14–4.72)	0.85 (0.07–10.71)	0.94 (0.13–6.79)	H + oNAC+SB + PTX	–	–	–	–
**0.33 (0.14–0.79)**	0.44 (0.06–3.26)	0.48 (0.06–3.70)	0.68 (0.13–3.45)	0.78 (0.15–4.12)	0.81 (0.08–8.40)	0.90 (0.16–5.06)	0.95 (0.08–10.69)	H + oNAC+sRIPC	–	–	–
0.33 (0.08–1.27)	0.43 (0.12–1.60)	0.47 (0.14–1.65)	0.66 (0.37–1.19)	0.77 (0.40–1.48)	0.80 (0.14–4.71)	0.88 (0.41–1.90)	0.94 (0.14–6.09)	0.98 (0.20–4.93)	H + iNAC	0.92 (0.30–2.83)	**0.71 (0.51–1.00)**
0.30 (0.08–1.11)	0.40 (0.11–1.39)	0.43 (0.12–1.61)	**0.61 (0.39–0.96)**	0.70 (0.40–1.24)	0.74 (0.13–4.16)	0.81 (0.39–1.68)	0.86 (0.14–5.44)	0.90 (0.19–4.34)	0.92 (0.63–1.35)	H + oNAC	**0.73 (0.58–0.93)**
**0.23 (0.06–0.88)**	0.31 (0.09–1.10)	0.34 (0.09–1.23)	**0.47 (0.29–0.77)**	**0.55 (0.31–0.97)**	0.57 (0.10–3.27)	0.63 (0.32–1.26)	0.67 (0.11–4.23)	0.70 (0.14–3.42)	**0.71 (0.52–0.99)**	**0.78 (0.62–0.97)**	H

Comparisons between treatments should be read from left to right. The results of both pooled effect sizes of the direct comparison (upper right side) and network meta-analysis (lower left side) are presented for the prevention efficacy of CA-AKI. The meta-analytic outcomes are expressed as OR with 95% confidence intervals for network analysis. OR less than 1 indicate that the prevention strategy of CA-AKI located in the row is more effective. Statistically significant findings are highlighted in bold. The CA-AKI prevention analysis encompasses data from 74 RCTs. CA-AKI, Contrast-associated acute kidney injury; FBX, Febuxostat; H, Hydration; iNAC, Intravenous form N-acetylcysteine; oNAC, Oral form N-acetylcysteine; OR, Odds ratio; PTX, Pentoxifylline; RCTs, Randomized controlled trials; RIPC, Remote ischemic preconditioning; sRIPC, Sham remote ischemic preconditioning; SB, Sodium bicarbonate; ST, statin.

In addition, we also conducted subgroup analyses targeting different populations, such as CKD, IOCM (iso-osmolar contrast media), LOCM (low-osmolar contrast media), and study era (conducted after 2010). For the CKD subgroup, we applied two commonly used thresholds according to the reporting in the original trials: (1) eGFR/creatinine clearance <45 mL/min or serum creatinine ≥1.45 mg/dL, and (2) eGFR/creatinine clearance <60 mL/min or serum creatinine ≥1.20 mg/dL. The detailed definitions and results are presented in Supplementary Figure S3.

### Assessment of consistency

A forest plot was generated to demonstrate odds ratio generated from direct and indirect pairwise comparisons using a random effects model. Effect estimates and confidence intervals are listed in Supplementary Figure S4.

We evaluated the transitivity assumption in Supplementary Figure S5 by analyzing the distributions of key effect modifiers, including age, baseline severity, and intervention duration, across the included studies. The current analysis does not suggest any violations of the transitivity assumption.

The comparison of these modifiers using the design-by-treatment interaction model revealed no significant differences between studies comparing different interventions (*p* = 0.32). However, when using the side-splitting model (Supplementary Table S4), we observed mild inconsistency between the hydration group and the hydration plus oral NAC group, as well as between the hydration group and the hydration plus oral NAC and statin group (*p* = 0.045). These findings suggest incoherence between direct and indirect evidence for these two comparisons.

### Assessment of GRADE

Supplementary Table S5 summarizes the evidence quality for the network meta-analysis comparisons, as assessed using the GRADE system. The quality of the evidence varies from very low to high.

## Discussion

This meta-analysis focusing on patients with cardiovascular angiography, highlights that the combination of hydration with oral or intravenous NAC is more effective than hydration alone in preventing CA-AKI. Furthermore, the addition of a statin to hydration with oral NAC provides even greater protection against CA-AKI compared to hydration with oral NAC alone.

Hydration with saline is a widely recognized strategy for preventing CA-AKI, particularly in patients undergoing cardiovascular angiography (4–9). Adequate hydration is effective in preventing CA-AKI because it reduces urine viscosity, which tends to increase with the volume of CM used (46). This correlation between hydration and reduced urine viscosity following CM administration has been validated through studies in both animals and humans (46, 47).

In addition, NAC is considered to prevent oxidative damage in CA-AKI primarily through its role as an antioxidant and a precursor to glutathione, a critical endogenous antioxidant (48, 49). After CM administration, the production of ROS increases, leading to oxidative stress, renal vasoconstriction, and cellular injury, contributing to CA-AKI pathogenesis (50). NAC helps mitigate these effects by boosting intracellular glutathione levels, neutralizing ROS, and protecting renal cells from oxidative damage (48). Moreover, NAC possesses vasodilatory properties that may enhance renal blood flow, further limiting ischemic damage from contrast exposure (48).

While NAC is believed to prevent CA-AKI through its antioxidant properties and ability to reduce oxidative stress, meta-analyses, especially those from larger trials, have not consistently supported this benefit (51). The positive effects reported in some studies are likely due to smaller trial sizes, specific outcome choices, and publication bias, with more reliable results observed when focusing on large-scale RCTs or trials measuring clinical outcomes rather than surrogate biomarkers (51).

The combined use of NAC and hydration appears to offer synergistic benefits, as hydration helps to dilute contrast media and reduce renal vasoconstriction, while NAC works to neutralize oxidative stress. This dual approach is particularly advantageous because hydration alone cannot fully address the oxidative stress caused by CM, and NAC alone may not provide sufficient renal protection without the volume expansion and hemodynamic support provided by hydration. A meta-analyses have shown that the combination of NAC and hydration reduces the risk of CA-AKI more effectively than NAC alone (52).

Statins not only lower low-density lipoprotein (LDL) cholesterol but also have pleiotropic effects, such as improving endothelial function, increasing nitric oxide availability, reducing inflammation, and stabilizing atherosclerotic plaques (29). In addition to their cardiovascular benefits, statins have been shown to reduce the risk of CA-AKI in patients with acute coronary syndrome, especially those with elevated baseline high-sensitivity C-reactive protein levels, offering kidney protection and improving short- and mid-term outcomes in this high-risk population (53). Additionally, combining high-dose statins with NAC and hydration has been shown to significantly reduce the risk of CA-AKI compared to hydration alone (34). This approach, supported by a systematic review and Bayesian network meta-analysis, highlights the complementary effects of statins and NAC in reducing oxidative stress and inflammation, providing a stronger preventive strategy for CA-AKI (34).

In the overall analysis using hydration alone as the reference, several combined strategies demonstrated significant protection against CA-AKI. Hydration plus oral NAC (OR 0.78, 95% CI 0.62–0.97) and hydration plus IV NAC (OR 0.71, 95% CI 0.52–0.99) were both superior to hydration alone, while the addition of statins to oral NAC and hydration provided an even greater benefit (OR 0.47, 95% CI 0.29–0.77) (Figure 3A). Consistent results were observed when oral NAC was used as the comparator, where hydration plus statin again showed significant protective effects (OR 0.61, 95% CI 0.39–0.96) (Figure 3B). Beyond statistical ranking, the clinical relevance of these findings is supported by absolute measures: the incidence of CA-AKI was reduced from 15.49% with hydration alone to 8.28% with hydration plus oral NAC and statin, corresponding to an absolute risk reduction of 7.2% and a number needed to treat of 14.

Subgroup analyses stratified by renal function further suggested that the benefit of adding statins was more pronounced in patients with advanced CKD. Using the broader threshold (eGFR <60 mL/min or serum creatinine ≥1.20 mg/dL, Supplementary Figure S3A), the combination of hydration, oral NAC, and statin showed a non-significant trend toward benefit. In contrast, applying the stricter threshold (eGFR <45 mL/min or serum creatinine ≥1.45 mg/dL, Supplementary Figure S3B) yielded a statistically significant risk reduction (OR 0.30, 95% CI 0.10–0.91). This gradient suggests that while the combination is effective across CKD stages, its absolute clinical benefit is maximized in high-risk patients with more advanced renal impairment.

The P-score ranking further indicated that combinations involving oral NAC with RIPC (0.85), febuxostat (0.76), or IV NAC with RIPC (0.72) had the highest probabilities of being the most effective strategies. Hydration combined with oral NAC and statin ranked in the middle range (0.63), yet it demonstrated robust protective effects in both the overall and subgroup analyses, with additional support from absolute risk reduction and number needed to treat estimates. These findings emphasize that P-scores should be interpreted with caution: while they provide a relative hierarchy, they do not directly translate into clinical applicability. Interventions such as RIPC or febuxostat, despite their high ranking, are supported by fewer trials and may be less feasible in routine practice. By contrast, statins are widely available, safe, and backed by a larger evidence base, making the combination of hydration, oral NAC, and statin a pragmatic and clinically meaningful preventive strategy despite its intermediate P-score.

Our finding that hydration plus IV NAC was associated with a modest reduction in CA-AKI differs from some previous meta-analyses reporting neutral effects. This discrepancy may be explained by differences in intervention protocols (e.g., NAC dosage and timing) and heterogeneity in AKI definitions across studies.

Another reason our findings may differ from prior meta-analyses is the handling of co-interventions. In many earlier analyses, hydration was provided as a background therapy but not explicitly acknowledged in the comparisons. As a result, reported contrasts such as “NAC vs. placebo” were effectively “hydration + NAC vs. hydration alone,” which may obscure the true incremental benefit of pharmacological agents beyond hydration. By explicitly modeling hydration as the common baseline intervention in our network, our study provides a more clinically meaningful comparison of combined strategies.

Current guidelines recommend adequate hydration as the cornerstone of CA-AKI prevention, while NAC and statins are not routinely endorsed due to inconsistent evidence. Our results suggest that the combination of hydration with NAC and statin may provide additional protection, highlighting an area where future guideline updates could consider integrating evidence from large-scale NMA. While our findings provide hypothesis-generating evidence for combined preventive strategies, the heterogeneity of existing trials underscores the need for well-designed, adequately powered RCTs to confirm these benefits before firm recommendations can be incorporated into clinical guidelines.

### Strength and limitation

Our study has several strengths. While hydration remains the most evidence-supported strategy for preventing CA-AKI, this analysis extends current knowledge by systematically evaluating the combined use of hydration with other pharmacological agents. By integrating these interventions into a single network, our study provides comparative effectiveness estimates and a treatment ranking, offering clinicians a broader range of evidence-based options for preventing CA-AKI in clinical practice.

However, there are also several limitations to our study. First, while we focused on intra-arterial procedures, which are commonly linked to CA-AKI, the procedures differed in the volume of contrast media administered. Second, although all fundamental prevention strategies were considered, lactated Ringer’s solution was excluded due to the limited number of studies available for the network meta-analysis, which could limit the comprehensiveness of the analysis. Third, any prevention strategy that did not include hydration was also excluded from the analysis, which may have limited the scope of potential interventions and influenced the findings. Fourth, the definition of AKI was not uniform across studies, which may have introduced heterogeneity and affected comparability. Fifth, mild inconsistency was observed between direct and indirect comparisons, particularly among strategies involving hydration ± NAC ± statins. This may reflect violations of the transitivity assumption due to differences in potential effect modifiers, such as baseline CKD severity, contrast volume, NAC/statin dose, and variations in hydration protocols. Finally, The coexistence of two widely accepted CA-AKI definitions may introduce heterogeneity across studies. We chose to include both in order to maximize generalizability, acknowledging that this may affect comparability. Since both definitions are commonly applied in clinical practice and research, we considered it reasonable to accept the reported outcomes without further stratification. These limitations suggest that while our findings provide useful comparative insights, clinical application should still be tailored to individual patient characteristics and procedural contexts.

## Conclusion

This network meta-analysis suggests that combining hydration with either oral or intravenous NAC may be more effective in reducing the risk of CA-AKI compared to hydration alone. Additionally, the use of a statin with hydration and oral NAC appears to offer better protection than hydration with oral NAC alone. However, it is important to note that the volume of contrast media varied across the included studies, and only saline-based hydration strategies were analyzed in this review. Further research is needed to confirm these findings and explore additional prevention strategies for CA-AKI.

## Data Availability

The datasets generated and/or analyzed during the current study are available from the corresponding author on reasonable request.

## Glossary

ACS: acute coronary syndrome
CA-AKI: contrast-associated acute kidney injury
CAG: coronary angiography
CAD: coronary artery disease
CKD: chronic kidney disease
CI: confidence interval
ClCr: creatinine clearance by Cockcroft-gault equation
CM: contrast media
eGFR: estimated glomerular filtration rate
EVAR: endovascular aneurysm repair
GRADE: grading of recommendations assessment, development and evaluation
H: hydration
H/S: Half saline
FBX: febuxostat
iNAC: intravenous form N-acetylcysteine
IPC: ischemic preconditioning
IQR: interquartile range
IV: intravenous
MeSH: Medical Subject Heading
NAC: N-acetylcysteine
LDL: low-density lipoprotein
oNAC: oral form N-acetylcysteine
OR: odds ratio
PA: peripheral angiography
PCI: percutaneous intervention
PRISMA: Preferred Reporting Items for Systematic Reviews and Meta-Analyses
PTCA: percutaneous transluminal coronary
RCT: randomized controlled trials
RIPC: remote ischemic preconditioning
ROS: reactive oxygen species
sRIPC: sham remote ischemic preconditioning
SB: sodium bicarbonate
ST: statin
