# Supplementary material for: Saline and N-acetylcysteine-based strategies and other approaches to prevent the risk of CA-AKI: a meta-analysis
Source: Front Med (Lausanne). 2025 Nov 12;12:1608626. doi: 10.3389/fmed.2025.1608626 (PMC12647111; doi:10.3389/fmed.2025.1608626)
Supplement: Supplementary file 1 [file Data_Sheet_1.pdf]

Saline and N-Acetylcysteine-Based Strategies and Other Approaches to Prevent the Risk of Contrast-Associated Acute Kidney Injury Among Patients Undergoing Cardiovascular Angiography: A Network Meta-Analysis

I-Chen Lin, Wen-Wen Tsai, Vin-Cent Wu, Heng-Chih Pan, Min-Hsiang Chuang, Jui-Yi Chen

Supplementary appendix

This supplementary appendix provides:

1. Search equation via PubMed, EMBASE and Cochrane library
2. PROSPERO protocol registration.
3. Table S1. Basic characteristic of included trials
4. Table S2. The number of each intervention within network
5. Figure S1. Risk of bias assessment
6. Figure S2. Funnel plot for the assessment of publication bias of the included studies in this network meta-analysis.
7. Figure S3A. Forest plot for different strategies to prevent the risk of CA-AKI among CKD patients.
   Figure S3B. Forest plot for different strategies to prevent the risk of CA-AKI among the patients with low osmolar contrast
   Figure S3C. Forest plot for different strategies to prevent the risk of CA-AKI among the patients with Iso-osmolar contrast
   Figure S3D. Forest plot for different strategies to prevent the risk of CA-AKI among the patients with contrast volume exceeding 120 mL
   Figure S3E. Forest plot for different strategies to prevent the risk of CA-AKI with the studies conducted after 2010
8. Table S3. P-score rankings of different interventions for preventing CA-AKI
9. Figure S4. Forest plot displaying the separation of direct and indirect evidence for interventions aimed at preventing CA-AKI, and assessing the consistency between each comparison within our network meta-analysis.
10. Figure S5: Scatter plot: distribution of treatment absolute effects on CA-AKI across different interventions
11. Table S4. Consistency test by side-splitting model
12. Table S5. GRADE in CA-AKI events across different intervention comparisons

1. **Search equation via PubMed, Cochrane and EMBASE library**
   A. The final search was undertaken on 26th January 2024
   B. Keyword of Pubmed

((randomized controlled trial[pt] OR controlled clinical trial[pt] OR randomized[tiab] OR randomized[tiab] OR placebo[tiab] OR clinical trials as topic[mesh:noexp] OR randomly[tiab] OR trial[ti] NOT (animals[mh] NOT humans [mh])))

AND

(Acetylcysteine[Mesh] OR N-Acetyl-L-cysteine OR N Acetyl L cysteine OR N-Acetylcysteine OR N Acetylcysteine OR Mercapturic Acid OR Acid, Mercapturic OR Solmucol OR Genac OR Acemuc OR Acetabs OR Acetylcystein AL OR NAC AL OR Acetylcystein Atid OR Acetylcystein Heumann OR Acetylcystein Trom OR Acetylcysteine Hydrochloride OR Hydrochloride, Acetylcysteine OR Acetylcysteine Sodium OR Sodium, Acetylcysteine OR Acetylcysteine, Monosodium Salt OR Monosodium Salt OR Acetylcysteine OR Acetylcysteine Zinc OR Zinc, Acetylcysteine OR Acetylcysteine, (D)-Isomer OR Acetylcysteine, (DL)-Isomer OR Acetylcysteine, Monoammonium Salt OR Monoammonium Salt Acetylcysteine OR Acetyst OR Acetylcysteine GNR OR Airbron OR Alveolex OR Bromuc OR Azubronchin OR Bisolvon NAC OR NAC, Bisolvon OR Broncho-Fips OR Broncho Fips OR BronchoFips OR Broncholysin OR Broncoclar OR Codotussyl OR Cystamucil OR Dampo Mucopect OR Mucopect, Dampo OR durabronchal OR Larylin NAC OR Eurespiran OR Exomuc OR Fluimucil OR NAC Zambon OR Zambon, NAC OR Fabrol OR Fluprowit OR Muco Sanigen OR Sanigen, Muco OR Frekatuss OR Jenacystein OR Jenapharm OR Lantamed OR Lindocetyl OR M-Pectil OR M Pectil OR MPectil OR mentopin OR Acetylcystein OR Acetylcystein, mentopin OR Muciteran OR Mucomyst OR Acetylin OR Mucosil OR Mucosol OR Mucosolvin OR Siccoral OR Siran OR Ilube OR Hoestil OR acebraus)

AND

((Cardiovascular[All Fields]) OR ("Cardiovascular System"[Mesh])) OR ((("Angiography"[Mesh]) OR (Angiographies[All Fields]) OR (Angiogram[All Fields] OR Angiograms[All Fields])) OR Arteriography[All Fields] OR Arteriographies[All Fields]) OR ("Angioplasty"[Mesh]) OR Angioplasties[All Fields] OR Percutaneous Transluminal Angioplasty[All Fields] OR Angioplasty, Percutaneous Transluminal[All Fields] OR Transluminal Angioplasty, Percutaneous[All Fields] OR Angioplasty, Transluminal[All Fields] OR Transluminal Angioplasty[All Fields] OR ("Percutaneous Coronary Intervention"[Mesh]) OR (Percutaneous Coronary Revascularizations[All Fields]) OR ("Cardiac Catheterization"[Mesh]) OR (Heart Catheterization[All Fields]) OR (Diagnostic Coronary Angiography[All Fields]) OR ("Stents"[Mesh])

AND

((Contrast) OR ("Contrast Media"[Mesh]))

AND

(((((((Kidney Diseases[All Fields]) OR ("Kidney Diseases"[Mesh])) OR (nephropathy)) OR ("Acute Kidney Injury"[Mesh])) OR ("acute kidney disease"[All Fields])) OR (Acute Kidney failure[All Fields])) OR (Acute Renal Insufficiency[All Fields]))

C. Cochrane library

#1 MeSH descriptor: [Contrast media] explode all trees
#2 MeSH descriptor: [Acute Kidney Injury] explode all trees
#3 MeSH descriptor: [Acetylcysteine] explode all trees
#4 MeSH descriptor: [Angioplasty, Balloon, Coronary] explode all trees
#5 (“contrast media”) (Word variations have been searched)
#6 (Acute Kidney injury) (Word variations have been searched)
#7 (Acetylcysteine) (Word variations have been searched)
#8 (Angioplasty, Balloon, Coronary) (Word variations have been searched)
#9 (#1 or #6) and (#2 or #7) and (#3 or #8) and (#4 or #9)

D. EMBASE

#14 # 12 AND #13
#13 'crossover procedure': de OR 'double-blind procedure':de OR 'randomized controlled trial':de OR 'single-blind procedure':de OR random*:de,ab,ti OR factorial*:de:ab,ti OR crossover:de,ab,ti OR ((cross NEXT/1 over):de;ab,ti) OR placebo*:de,ab,ti OR ((doubl* NEAR/1 blind*):de,ab,ti) OR ((singl* NEAR/1 blind*):de:ab,ti) OR assign*:de,ab,ti OR allocat*:de,ab,ti OR volunteer:de,ab,ti
#12 # 1 AND #7 AND #10 AND #11
#11 #8 OR #9
#10 #2 OR #3 OR #4 OR #5 OR #6
#9 'acute kidney failure'/exp OR 'acute kidney failure'
#8 'kidney disease'/exp OR 'kidney disease'
#7 'contrast/exp OR 'contrast*
#6 'stent/exp OR 'stent*
#5 'heart catheterization'/exp OR 'heart catheterization'
#4 'percutaneous coronary intervention'/exp OR 'percutaneous coronary intervention'
#3 'angioplasty'/exp OR 'angioplasty'
#2 'angiography'/exp OR 'angiography'
#1 'acetylcysteine'/exp OR 'acetylcysteine’

2. PROSPERO protocol registration

1. * Review title.

Series of N-acetylcysteine plus saline based hydration with or without additional treatments and acute kidney injury after coronary angiography or percutaneous coronary intervention: A Network Meta-analysis

2. Original language title.

Series of N-acetylcysteine plus saline based hydration with or without additional treatments and acute kidney injury after coronary angiography or percutaneous coronary intervention: A Network Meta-analysis

3. * Anticipated or actual start date.

15/01/2024

4. * Anticipated completion date.

19/02/2024

5. * Stage of review at time of this submission.

The review has not yet started: No

Review stage	Started	Completed
Preliminary searches	Yes	No
Piloting of the study selection process	Yes	No
Formal screening of search results against eligibility criteria	Yes	No
Data extraction	No	No
Risk of bias (quality) assessment	No	No
Data analysis	No	No

Provide any other relevant information about the stage of the review here.

6. * Named contact.

I Chen Lin

Email salutation (e.g. "Dr Smith" or "Joanne") for correspondence:

Mr Lin

7. * Named contact email.

a8888588885@gmail.com

8. Named contact address

No. 158, Sec. 4, Ximen Rd., North Dist., Tainan City 704 , Taiwan (R.O.C.)

9. Named contact phone number.
886-983703905

10. * Organisational affiliation of the review.
Division of Nephrology, Department of Internal Medicine, Chi Mei Medical Center, Tainan, Taiwan

Organisation web address:
http://www.chimei.org.tw/main/cmh_department/54220/english/

11. * Review team members and their organizational affiliations.
Mr. I Chen Lin, Department of Internal Medicine, Chi Mei Medical Center, Tainan, Taiwan

Mr. Jui-Yi Chen. Division of Nephrology, Department of Internal Medicine, Chi Mei Medical Center, Tainan, Taiwan

Mr. Wen-Wen Tsai, Department of Neurology, Chi-Mei Medical Center, Tainan, Taiwan

12. * Funding sources/sponsors.
nil
Grant number(s)
nil

13. * Conflicts of interest.
None

14. Collaborators.
nil

15. * Review question.
In adult patients undergoing Coronary Angiography or Percutaneous Coronary Intervention, does saline-based hydration plus N-acetylcysteine (NAC) with or without additional treatments compared to saline-based hydration alone reduce the risk of acute kidney injury?

16. * Searches.
PubMed, Embase, Cochrane

17. URL to search strategy.

Do not make this file publicly available until the review is complete

18. * Condition or domain being studied.

risk of CA-AKI for adults after Coronary Angiography(CAG) or Percutaneous Coronary Intervention(PCI)

19. * Participants/population.

Adult patients who underwent Coronary Angiography or Percutaneous Coronary Intervention

20. * Intervention(s), exposure(s).

saline based hydration plus NAC with or without additional treatments

21. * Comparator(s)/control.

Where relevant, give details of the alternatives against which the intervention/exposure will be compared (e.g., another intervention or a nonexposed control group). The preferred format includes details of both inclusion and exclusion criteria.

saline based hydration

22. * Types of study to be included.

Give details of the study designs (e.g., RCT) eligible for inclusion in the review. The preferred format includes both inclusion and exclusion criteria. If there are no restrictions on the types of study, this should be stated.

MEDLINE, Embase, and Cochrane Central Register of Controlled Trials databases for randomized clinical trials (RCTs) published from the database inception until January 2024.

23. Context.

Give summary details of the setting or other relevant characteristics, which help define the inclusion or exclusion criteria.

Prevention strategies for CAAKI in adult patients who underwent Coronary Angiography or Percutaneous Coronary Intervention

24. * Main outcome(s).

Give the pre-specified main (most important) outcomes of the review, including details of how the outcome is defined and measured and when these measurements are made if these are part of the review inclusion criteria.

risk of CAAKI

* Measures of effect

Please specify the effect measure(s) for your primary outcome(s) e.g., relative risks, odds ratios, risk difference, and/or 'number needed to treat.

odds ratios

25. * Additional outcome(s).

List the pre-specified additional outcomes of the review, with a similar level of detail to that required for main outcomes. Where there are no additional outcomes, please state 'None' or 'Not applicable' as appropriate to the review

nil

* Measures of effect

Please specify the effect measure(s) for your additional outcome(s) e.g., relative risks, odds ratios, risk difference, and/or 'number needed to treat.

nil

26. * Data extraction (selection and coding).

Describe how studies will be selected for inclusion. State what data will be extracted or obtained. State how this will be done and recorded.

The following data were extracted from the full-text articles: the first author name, year of publication, sample size, study design, patient inclusion criteria, patient demographics, clinical outcome, and adverse events.

27. * Risk of bias (quality) assessment.

State which characteristics of the studies will be assessed and/or any formal risk of bias/quality assessment tools that will be used.

The RoB 2.0 assessment for individually randomized trials (including cross-over trials) has five domains, as
follows.
(1) Bias arising from the randomization process.
(2) Bias due to deviations from intended interventions.
(3) Bias due to missing outcome data.
(4) Bias in measurement of the outcome.
(5) Bias in selection of the reported result.

28. * Strategy for data synthesis.

Describe the methods you plan to use to synthesize data. This **must not be generic text** but should be **specific to your review** and describe how the proposed approach will be applied to your data. If meta-analysis is planned, describe the models to be used, methods to explore statistical

heterogeneity, and software package to be used.
Between-trial heterogeneity was determined by using I^2 tests, and values > 50% were regarded as considerable heterogeneity.

29. * Analysis of subgroups or subsets.
Nil

30. * Type and method of review.
Type of review
Cost-effectiveness: No
Diagnostic: No
Epidemiologic: No
Individual patient data (IPD) meta-analysis: No
Intervention: No
Meta-analysis: No
Methodology: No
Narrative synthesis: No
Network metaanalysis: Yes
Pre-clinical: No
Prevention: No
Prognostic: No
Prospective meta-analysis (PMA): No
Review of reviews: No
Service delivery: No
Synthesis of qualitative studies: No
Systematic review: Yes
Other: No

Health area of the review
Alcohol/substance misuse/abuse: No
Blood and the immune system: No
Cancer: No
Cardiovascular: No
Care of the elderly: No
Child health: No
Complementary therapies: No
COVID-19: No
Crime and justice: No
Dental: No

Digestive system: No
Ear, nose, and throat: No
Education: No
Endocrine and metabolic disorders: No
Eye disorders: No
General interest: No
Genetics: No
Health inequalities/health equity: No
Infections and infestations: No
International development: No
Mental health and behavioral conditions: No
Musculoskeletal: No
Neurological: No
Nursing: No
Obstetrics and gynecology: No
Oral health: No
Palliative care: No
Perioperative care: No
Physiotherapy: No
Pregnancy and childbirth: No
Public health (including social determinants of health): No
Rehabilitation: No
Respiratory disorders: No
Service delivery: No
Skin disorders: No
Social care: No
Surgery: No
Tropical Medicine: No
Urological: No
Wounds, injuries, and accidents: No
Violence and abuse: No

31. Language.

English
There is not an English language summary

32. * Country.

Taiwan

33. Other registration details.

none

34. Reference and/or URL for published protocol.

No, I do not make this file publicly available until the review is complete

35. Dissemination plans.

No

36. Keywords.

Contrast induced acute kidney injury(CI-AKI), contrast associated acute kidney injury(CAAKI), N-acetylcysteine(NAC)

37. Details of any existing review of the same topic by the same authors.

none

38. * Current review status.

Review ongoing

39. Any additional information.

none

40. Details of final report/publication(s) or preprints if available.

none

Table S1. Basic characteristic of included trials

The table cells shaded in gray indicate exclusion from this analysis due to failure to meet inclusion criteria.

Study (year)	Procedure	Osmolarity	Basic strategy	Intervention	Number of group	Number of CA-AKI	Mean age (year)	Male n (%)	Female n (%)	Dose	Contrast Volume (ml)	Baseline kidney function
Sarhan et al. (2023)	PCI	Low	N/S + oral NAC	Febuxostat	60	7	61.07±7.3*	32(53.3)	28(46.7)	H+NAC, also received Febuxostat 80 mg within 6–18 h before PCI and 6–18 h after PCI with a time gap of 24 h between the two doses.	185.83±41.6*	1.59±0.19** (mg/dL)
				control	60	15	58.39±7.2	32(53.3)	28(46.7)	H+NAC	190.174±42.6*	1.64±0.27** (mg/dL)
Sahu et al. (2021)	CAG	Low	N/S+oral NAC	RIPC	45	2	57.76±7.8	38(84.4)	7(15.6)	IPC was accomplished by performing 4 cycles of alternating 5-min inflation and 5-min deflation of a standard upper-arm blood pressure cuff to the individual's systolic blood pressure plus 50 mmHg to induce transient and repetitive arm	40.02±7.61*	2.16±0.46** (mg/dL)

										ischemia and reperfusion. IPC was started immediately before CAG. The time interval between the last inflation cycle and the start of CAG was <45 min.		
				Control	42	8	55.35±10.76	30(71.4)	12(28.6)	Sham preconditioning was performed in the same way as IPC, by inflating an upper-arm blood pressure cuff to diastolic pressure levels and then deflating the cuff for 10 mmHg to maintain nonischemic upper-arm compression for blinding purposes with regard to the patients.	37.04±6.04*	2.14±0.44** (mg/dL)
Samadi et al. (2020)	CAG	Iso	N/S	Control	72	0	62.98±9.2	33(45.8)	39(54.2)	N/S infusions 1 ml/kg from 12	NR	1.31±0.33** (mg/dL)

										hours before CAG till 12 hours after it		
				oral NAC	66	0		20(30.3)	46(69.7)	N/S and oral NAC (1200 mg/twice a day) from 24 hours before CAG to the day of CAG	NR	1.21±0.18** (mg/dL)
				α-tocopherol (excluded)	63	0		21(33.3)	42(66.7)	Excluded from analysis		
Stokfisz et al. (2020)	CAG or PCI	Low	N/S + oral NAC	RIPC	50	2	66	NR	NR	a cuff was placed on the upper left arm and 4 cycles comprising a 5-minute inflation to 200 mm Hg followed by a 5-minute deflation were performed (contralaterally to subsequent further radial catheters placement). The RIPC protocol began within 1 hour before CAG or PCI, and was completed before the start of the procedure. The time	Median: 100	87(81-96)# (umol/L)

										between the end of the last inflation of the blood pressure cuff and the placement of the right radial PCI catheter was < 25 minutes.		
				Control	51	3	65	NR	NR	A deflated cuff was placed on the left arm for 40 minutes.	Median: 110	88 (82-101)[#] (umol/L)
Aslanabadi et al. (2019)	PA	Iso	N/S + NaHCO3 + oral NAC	PTX	40	4	62.9±10	16(40)	24(60)	1200-mg dose of PTX orally (3 tablets, 400 mg) 2–4 h before PA based on the time to peak of PTX plus the standard treatment	150±20[*]	1.02±0.19[**] (mg/dL)
				Control	36	3	58.3±7.3	23(63.9)	13(36.1)	Only standard treatment	150±20[*]	1.01±0.17[**] (mg/dL)
Barzi et al. (2019)	CAG or PCI	Iso	N/S + oral NAC	PTX	55	3	70.1±11.8	23(41.8)	32(58.2)	400 mg of PTX three times per day from 24 h before to 48 h after the procedure	NR	1.31±0.81[**] (mg/dL)
				Control	55	4	68.3±10.83	29(52.7)	26(47.3)	placebo	NR	1.2±0.33[**] (mg/dL)
Biernacka-	CAG or PCI	Low/Iso	N/S	IV NAC	108	9	66±8.9	88(81.5)	26(18.5)	IV NAC 600 mg, diluted in 100 ml of N/S,	196.7±85.9[*]	Mean eGFR. ±SD: 98.5±42

Fialkowska et al. (2018)										on the day before the procedure (about 12 h before), on the day of administration of the CM twice daily, and on the day after administration of the CM twice daily (5 doses, of which 2 were given before and 3 were given after exposure to CM).		ml/min/1.73m 2
				Control	114	21	64.3±9.5	83(72.8)	31(27.2)	100 ml of N/S	204±84.2*	Mean eGFR. ±SD: 94.3±36.3 ml/min/1.73m 2
Ghaemian et al. (2018)	CAG or PCI	Iso	N/S + oral NAC	RIPC	66	2	66.15±8.63	51(77.3)	15(22.7)	A standard blood pressure cuff to the patients' upper arms. RIPC was performed with four cycles of 5-min inflation, followed by 5-min deflation of the cuff at 50 mmHg above the patient's	Median (IQR): 90 (80–112.5)	Mean eGFR. ±SD: 41.86±8.16 ml/min/1.73m 2

									systolic blood pressure.		
				control	66	4	65.27±8.9	42(63.6)	24(36.4)	The four cycles of 5-min inflation and deflation of the blood pressure cuff were performed, but only at10 mmHg below the patient's diastolic pressure.	Median (IQR): 100 (80–116)	Mean eGFR. ±SD: 42.77±9.46 ml/min/1.73m2
Shalaby et al. (2018)	CAG or PCI	NR	N/S + oral NAC	Atorvastatin	150	14	54.45±12.99	78(52)	72(48)	80mg atorvastatin, 12 h before the procedure, with a further 40 mg pre-procedural dose	NR	0.95±0.24** (mg/dL)
				Control	150	30	55.32±12.93	81(54)	69(46)	placebo	NR	0.96±0.26** (mg/dL)
Valappil et al. (2018)	PCI	Low	N/S + oral NAC	RIPC	50	11	62.8±9.1	43(86)	7(14)	4 cycles of alternating 5 min inflations and 5 min deflations using a standard upper-arm blood pressure cuff to a level 50 mm Hg above the individual's systolic blood pressure to	218.6	#1.5(1.3-1.7) (mg/dL)

										induce transient arm ischemia followed by reperfusion. RIPC was started immediately before cardiac catheterization. The time between the last inflation cycle and the start of procedure was <45 min.		
				Control	50	18	60.5±8.5	40(80)	10(20)	Sham conditioning was done by inflating the blood pressure cuff to 30 mm of Hg for 5 min followed by deflation for 5 min for a total of 4 cycles.	198.4	1.4(1.4-1.6)[#] (mg/dL)
Wojciechowska et al. (2018)	PCI	Low	N/S+IV NAC	RIPC	61	3	64.4±10.5	41(67.2)	20(32.8)	three cycles of 5-min inflation to 200 mmHg and 5-min deflation of a standard upper arm blood pressure cuff. The time	155.5±76.7[*]	0.86±0.27[**] (mg/dL)

										between the last inflation cycle and PCI was <2 h.		
				Control	58	7	62.9±8.6	39(67.2)	19(32.8)	Blood pressure measured only 2 h before PCI.	143.7±55.7*	0.86±0.26** (mg/dL)
Syed et al. (2017)	CAG or PCI	NR	N/S + oral NAC	Atorvastatin	80	2	51.87±8.48	52(65)	28(35)	Atorvastatin 80 mg + NAC 1200 mg once daily, for 3 days before and 2 days after the procedure	NR	1.152±0.124** (mg/dL)
				Control	80	9	53.12±7.55	47(58.8)	33(41.2)	Oral NAC 1200 mg once daily	NR	124±0.128** (mg/dL)
Sadineni et al. (2017)	CAG or PCI	Iso	N/S	oral NAC	35	7	60.74±10.61	27(77.1)	8(22.9)	Oral NAC 600 mg twice daily, the day before and the day of the procedure. N/S was infused at a rate of 0.5 ml/kg/h 12 h prior to the procedure and was continued for 12 h after contrast administration (total 24 h).	61.4±34.8*	2.24±0.9*** (mg/dL)
				Allopurinol (excluded)	30	5	62.9±8.67	23(76.7)	7(23.3)	Excluded from analysis		
				Control	30	11	62.6±11.84	26(86.7)	4(13.3)	N/S only	77.33±43.30*	2.19±1.01** (mg/dL)

Khosravi et al. (2016)	CAG	Iso	N/S + oral NAC	Atorvastatin	110	3	63.85±8.88	NR	NR	Atorvastatin 80mg per day from 48h before the procedure	< 200 ml for 96.3% of patients	1.53±0.44** (mg/dL)
				Control	110	6		NR	NR	Matching Placebo	< 200 ml for 98.2% of patients	1.47±0.42** (mg/dL)
Pezeshgi et al. (2015)	CAG	Low	N/S + oral NAC	oral NAC	75	1	NR	67(44.6)	83(55.4)	Oral NAC 600 mg twice a day, one day before CAG and continued until the second day after CAG. Unclear N/S protocol.	NR	NR
				Control	75	13	NR			N/S only	NR	NR
Erturk et al. (2014)	CAG, PCI or PA	Low	N/S	IV NAC	102	13	66±9	66(64.7)	36(35.3)	IV NAC 2400mg 1 hour pre-procedure and 4800mg 4-6 hours post-procedure. N/S was administered at a rate of 1ml/kg/h for 12h before and 12h after the procedure.	122±67*	eGFR 30-59ml: n=97 eGFR 15-29ml: n=5
				Oral NAC	102	14	65±8	64(62.7)	38(37.3)	N/S. Oral NAC 1200 mg every 12 hours for 24 hours pre- and	127±89*	eGFR 30-59ml: n=95 eGFR 15-29ml: n=7

										48 hours post-procedure.		
				Control	103	7	67±8	65(63.1)	38(36.9)	N/S only	127±66*	eGFR 30-59ml: n=92 eGFR 15-29ml: n=11
Inda-Filho et al. (2014)	CAG	High	N/S	IV NAC	126	49	59.2±11.4	78(61.9)	48(38.1)	IV NAC was given at 150 mg/kg/hr one hour before CAG, then at 50 mg/kg/h during and 6 hours after CAG.<br>N/S was given at 1 ml/kg/hr at the same time.	NR	1.00±0.25** (mg/dL)
				NaHCO3 (excluded)	125	75	59.1±13	70(56)	55(44)	Excluded from analysis		
				Control	125	61	60.5±11.3	73(58.4)	52(41.6)	N/S only	NR	1.04±0.41** (mg/dL)
				Both NAC ± NaHCO3 (excluded)	124	72	58.6±10.8	82(66.1)	42(33.9)	Excluded from analysis		
Mahmoodi et al. (2014)	PCI	Low	N/S + oral NAC	NaHCO3	175	12	64.96±10.29	76(43.4)	99(56.6)	NaHCO3 solution was prepared by adding 154 ml of 1000 mEq/L NaHCO3 to 846 ml of 5% dextrose with water.<br>All the patients received a fixed	NR	1.16±0.4** (mg/dL)

										dose of fluid 6 h before the procedure and 6 h after it.		
				NaCl	175	34	64.48±11.07	104(59.4)	71(40.6)	1000ml N/S	NR	1.17±0.4** (mg/dL)
Kumar et al. (2014)	PCI	Low/Iso	N/S	Oral NAC	90	18	NR	NR	NR	600 mg orally twice daily and N/S 1ml/kg/hr (max of 100), 12h pre- and post- contrast exposure	NR	Omnipaque: 1.0 (0.9-1.3)# Visipaque 1.1 (0.9-1.2) (mg/dL)
				Allopurinol (excluded)	95	15	NR	NR	NR	300 mg orally 12h pre- and post- contrast and N/S		
				Control	90	31	NR	NR	NR	N/S only		
Leoncini et al. (2014)	PCI	Iso	N/S + oral NAC	Rosuvastatin	252	17	66.2±12.4	166(65.9)	86(34.1)	40 mg Rosuvastatin on time of randomization followed by 20 mg/day, N/S 1 ml/kg/hr 12 grs both before and after the procedure, oral NAC 1200mg twice a day form the day before through the day after the procedure	183±80*	0.95±0.27** (mg/dL)

				Control	252	38	66.1±13.5	165(65.5)	87(34.5)	N/S+oral NAC	127±72*	0.96±0.28** (mg/dL)
Thayssen et al. (2014)	CAG or PCI	Iso	N/S	Oral NAC	176	32	63(55-70.8)	127(72.2)	49(27.8)	N/S+NAC 1200 mg orally before the PCI followed by 1200 mg daily during the next 48 hours,	Median (IQR): 140(110-180)	0.84(0.71-0.97)#
				NaHCO3 (excluded)	181	33	62(52-75)	139	42	Excluded from analysis		
				NaHCO3+Oral NAC	177	33	63(53.5-73)	139(78.5)	38(21.5)	N/S+NAC+NaHCO3	Median (IQR): 140(110-180)	0.88(0.74-1.00)#
				Control	181	43	63(55-72)	145(80.1)	36(19.9)	N/S alone	Median (IQR): 150(110-180)	0.87(0.74-1.03) #
Yang et al. (2014)	CAG or PCI	Low	N/S	NaCl	161	5	69.66±11.08	86(54.8)	75(47.2)	N/S at 1.5mL/kg/h 6h pre-procedure, and continued for 6h after	124±63.81*	Mean eGFR. ±SD: 93.46±22.45 ml/min/1.73m2
				NaHCO3 (excluded)	159	8	58.71±10.98	84	75	Excluded from analysis		
				NaCl+oral NAC	157	7	57.83±11.54	86(54.8)	71(45.2)	N/S plus 600mg NAC twice daily 24h pre- and post-procedure	129±46.77*	Mean eGFR. ±SD: 93.84±21.98 ml/min/1.73m2
				NaHCO3+oral NAC	150	8	60.03±10.12	82(54.7)	68(45.3)	1.5 % NaHCO3 at 1.5 mL kg/h for 6h pre-procedure, continuing for 6h after plus 600mg NAC twice daily 24h	126±57.97*	Mean eGFR. ±SD: 92.76±23.05 ml/min/1.73m2

										pre- and post-procedure		
Albabtain et al. (2013)	CAG or PCI	Low	N/S	Oral NAC	62	5	62.0±9.4	44(71)	18(29)	600 mg orally twice daily for 2 days, starting the evening before the procedure	70.1±60.4*	1.45±0.56** (mg/dL)
				Ascorbic acid (excluded)	57	2	58.7±11.9	38	19	Excluded from analysis		
				Placebo	66	5	59.8±10.8	54(81.8)	12(18.2)	N/S with placebo	*97.4±99.4*	1.22±0.40** (mg/dL)
Berwanger et al. (2013)	CAG or PCI	Low/Iso/High	N/S	Oral NAC	702	97	64.6±9.9	421(60)	281(40)	1200 mg orally every 12 hours, 2 doses pre- and 2 doses post-procedure	Median (IQR): 100 (70-130)	1.1±0.5** (mg/dL)
				Placebo	667	98	64.3±9.9	391(58.6)	276(41.4)	N/S with Placebo	Median (IQR): 100 (70-130)	1.1±0.6** (mg/dL)
Brueck et al. (2013)	CAG or PCI	Low	N/S	IV NAC	192	53	75	130(67.7)	69(32.3)	600 mg in 250 ml N/S over 30 min 24 hours and one hour before contrast exposure	Median (IQR): 110 (80-160)	1.5 (1.3-1.8)# (mg/dL)
				Ascorbic acid (excluded)	98	24	75	65	37	Excluded from analysis		
				Control	193	62	74	123(61.1)	75(38.9)	N/S only	Median (IQR): 110 (80-150)	1.5 (1.3-1.7)# (mg/dL)
Aslanger et al. (2012)	CAG or PCI	Low	N/S	IV NAC	108	27	56.1±12	86(79.6)	22(20.4)	1200mg bolus during the procedure and 1200mg orally, twice daily for	193±57*	0.9±0.3** (mg/dL)

										48h post-procedure N/S was given to all groups for 12 h at a rate of 1 ml/kg/h.		
				Intra-renal NAC (excluded)	105	24	55.9±13	82	23	Excluded from analysis		
				Placebo	99	23	57.2±12	73(73.7)	26(26.3)	N/S+placebo	204±67*	0.86±0.3* (mg/dL)
Er et al. (2012)	CAG	Low	N/S+oral NAC	RIPC	50	6	73.2±9.1	34(68)	16(32)	Intermittent arm ischemia through 4 cycles of 5-minute inflation and 5-minute deflation of a blood pressure cuff started immediately before procedure.	124±44*	1.63 (1.47–1.81)# (mg/dL)
				Placebo	50	20	72.7±11.4	37(74)	13(26)	Sham preconditioning was performed by the same way, inflating an upper arm blood-pressure cuff to diastolic pressure levels and then deflating the cuff for 10	103±41*	1.62 (1.39–1.93)# (mg/dL)

									mmHg to maintain non-ischemic upper arm compression for blinding purposes of the patients.		
Gunebakmaz et al. (2012)	CAG or PCI	Low	N/S	oral NAC	40	9	64.7±11.9	29(72.5)	11(27.5)	600 mg orally twice daily for 4days, 4 doses pre-procedure, 2 doses day of procedure and 2 doses day post-procedure. N/S was given at a rate of 1 mL/kg/hour, for 6 hours before and 12 hours after the procedure.	Mean: 63.4	1.42±0.13** (mg/dL)
				Nebivolol (excluded)	40	8	64.1±9.0	29	11	Excluded from analysis		
				Control	40	11	66.4±10.7	25(62.5)	15(37.5)	N/S only	Mean: 64.2	1.43±0.14** (mg/dL)
Jaffery et al. (2012)	CAG or PCI	Iso	N/S	IV NAC	206	33	65.6±12.9	138(67)	68(33)	1,200 mg IV NAC (6g in in 500 ml of 5% dextrose solution in water) bolus followed by 200 mg/h for 24 hr.	169.5±94.5*	1.09±0.4** (mg/dL)

										N/S was given at 1 cc/kg/h for 24 hr.		
				Placebo	192	25	65.1±12.7	114(59.4)	78(40.6)	N/S only with placebo	161.3±83.4*	1.07±0.4** (mg/dL)
Koc et al. (2012)	CAG or PCI	Low	N/S	IV NAC	80	2	62±10	61(76.3)	19(23.7)	IV bolus of 600 mg twice daily before and on the day of procedure (total=2.4 g) plus N/S 1 ml/kg/h before, on and after the day of procedure	Median (IQR): 130 (100-155)	Mean CrCl. ±SD: 59±16 mL/min
				Control (excluded)	60	6	64±10	21	39	Excluded from analysis		
				NaCl	80	13	65±11	63(78.8)	17(21.2)	N/S only	Median (IQR): 120 (100-150)	Mean CrCl. ±SD: 58±16 mL/min
Saitoh et al. (2011)	CAG	Low	N/S	oral NAC	7	1	76.5±2.8	6(85.7)	1(14.3)	704 mg twice daily orally from 1 day pre-procedure for a total of 2 days. All patients received N/S at 1 ml/kg/h for 24 h that started at 12 h before the administration of contrast medium and was continued until 12 h after CAG.	117.1±9.0*	Mean CrCl. ±SD: 42.8±4.0 mL/min

				Control	7	1	72.1±2.7	6(85.7)	1(14.3)	N/S only	113.6±14.5*	Mean CrCl. ±SD: 36.6±4.1 mL/min
				Glutathione (excluded)	7	0	71.1±3.3	7	0	Excluded from analysis		
A.C.T Investigators (2011)	CAG, PCI or PA	Low/Iso/High	N/S	oral NAC	1172	147	68.0±10.4	727(62)	445(38)	1200 mg orally every 12 hours, 2 doses pre- and 2 doses post-procedure. N/S was given at 1 mL/kg per hour, from 6 to 12 hours before to 6 to 12 hours after angiography.	Median (IQR): 100 (70-130)	Mean SCr. 1.2±0.5(mg/dL)
				Placebo	1136	142	68.1±10.4	689(60.7)	447(39.3)	N/S with placebo	Median (IQR): 100 (70-130)	Mean SCr. 1.2±0.5(mg/dL)
Sadat et al. (2011)	PA	Low	N/S	oral NAC	21	1	NR	NR	NR	600 mg twice daily on the day pre- and 600 mg twice on the day of procedure. N/S 1 L 12 hours before angiography and 1 L over 12 hours following the procedure.	70±20*	97 (72-125)# (umol/L)
				Control	19	3	NR	NR	NR	N/S only	75±25*	88 (68-142)# (umol/L)
	CAG	Low	H/S	IV NAC	39	2	NR	31(79.5)	8(20.5)	600 mg in 50 ml saline (0.9%)	134.79±13*	2.01±0.77** (mg/dL)

Carbonell et al. (2010)										over 30 min twice daily for total of 4 doses, starting at least 6 hours pre-procedure. H/S was administered at a rate of 1 mL/Kg/h at the first 6 h before the procedure and was maintained for 12 h after it.		
				Control	42	10	NR	34(81)	8(19)	H/S only	184.66±21*	1.87±0.7** (mg/dL)
Kinbara et al. (2010)	CAG or PCI	Low	N/S	oral NAC	15	0	70±10	9(60)	6(40)	704 mg orally twice daily for 2 days, the day preceding and day of procedure. N/S was given intravenously at a rate of 1 ml/kg body weight per hour for 30 min before and 10 h after angiography.	147±23*	1.00±0.36** (mg/dL)
				Control	15	4	70±8	9(60)	6(40)	N/S only	141±14*	0.97±0.29** (mg/dL)

				Aminophylline (excluded)	15	0	71±3	10	5	Excluded from analysis		
Ozhan et al. (2010)	CAG	Iso	N/S+oral NAC	Statin	60	2	55±8	37(61.7)	23(38.3)	80 mg atorvastatin plus 600 mg NAC twice daily on day of procedure followed by 80 mg atorvastatin for 2 days after the procedure. All the patients were hydrated with standard 1000 mL saline infusion during 6 hours after the procedure.	97±7*	0.88±0.2** (mg/dL)
				Control	70	7	54±10	40(57.1)	30(42.9)	N/S and oral NAC	93±6*	0.88±0.19** (mg/dL)
Thiele et al. (2010)	CAG	Low	N/S	IV NAC	126	18	68	89(70.6)	37(29.4)	Intravenous bolus of 1,200 mg before angioplasty and 1,200 mg intravenously twice daily for 48 h after (total dose 6,000 mg). N/S infusion at a rate of 1 ml/kg of body weight per h for 12 h.	Median (Range): 180 (140-230)	81 (69-97)# (umol/L)

				Placebo	125	25	68	82(65.6)	43(34.4)	matched times - 10 ml of 0.9% NaCl at each injection	Median (Range): 160 (120-220)	78 (67-90)[#] (umol/L)
Toso et al. (2010)	CAG	Iso	N/S+oral NAC	Statin	152	15	75±8	104(68.4)	48(31.6)	80mg Atorvastatin daily starting 48h pre-procedure, continued for 48h post-procedure. N/S 1 ml/kg/h for 12 hours before and after the procedure	164±99[*]	1.2±0.35[**] (mg/dL)
				Placebo	152	16	76±7	92(60.5)	60(39.5)	N/S and matched placebo	151±95[*]	1.18±0.33[**] (mg/dL)
Amini et al. (2009)	CAG	Low/Iso	N/S	oral NAC	45	5	63.25±9.78	20(44.4)	25(55.6)	600 mg orally twice daily, starting the day before the procedure and continuing for 2 doses post-procedure. 1 L of N/S was given during the procedure.	118.00±35.20[*]	1.736±0.42[**] (mg/dL)
				Placebo	45	6	65.09±9.40	34(75.6)	11(24.4)	N/S and matched placebo	121.11±43.95[*]	1.736±0.17[**] (mg/dL)
Baskurt et al. (2009)	CAG	Low	N/S	oral NAC	73	7	67.9±9.9	46(63)	27(37)	600 mg orally twice daily day preceding and	115.61±35.2[*]	1.39±0.24[**] (mg/dL)

										day of angiography. N/S (1 m/kg/h) for 12 h before and after contrast exposure.		
				oral NAC+Theophylline (excluded)	72	0	67.1±10.7	43(59.7)	29(40.3)	N/S plus oral NAC and theophylline (200 mg oral theophylline twice daily for the preceding day and the day of angiography	130.69±44. *5	1.47±0.27** (mg/dL)
				Control	72	5	67.1±8.6	41(56.9)	31(43.1)	N/S only	113.54±37.7*	1.30±0.20** (mg/dL)
Ferrario et al. (2009)	CAG, PCI or PA	Iso	N/S	oral NAC	99	8	75±7.7	67(67.7)	32(32.3)	600 mg orally every 12 hours on day pre- and day of procedure. N/S 1 ml/kg/h in the 12–24 h before the procedure and in the following 24 h.	180±104.4*	Mean CrCl. ±SD: 37±11.5 ml/min
				Placebo	101	6	75±6.9	63(62.4)	38(37.6)	N/S and matched placebo	168±103.3*	Mean CrCl. ±SD: 40±9.3 ml/min
	CAG or PCI	Iso	Nil	NaCl	15	1	64±10	9(60)	6(40)	NaHCO3 (154 mL of 1000	131±63*	116.69±39.78** (umol/L)

Ratcliffe et al. (2009)				NaCl + oral NAC	21	1	65±11	11(52.4)	10(47.6)	mEq/L NaHCO3 to 846 mL of 5% dextrose) or NaCl (154 mEq/L NaCl in 5% dextrose), at an infusion rate of 3 mL/kg/h for 1 h before contrast, and continued at 1 mL/kg/h during the procedure and for 6 h following contrast exposure. NAC regimens received an intravenous bolus of 1200 mg of NAC 1 h before intervention and 1200 mg orally twice daily for 48 h after intervention.	175±81*	103.43±35.36** (umol/L)
				NaHCO3 (excluded)	19	2	67±11	11	8	Excluded from analysis		
				NaHCO3 + oral NAC (excluded)	23	1	65±12	16	7	Excluded from analysis		
Izani et al. (2008)	CAG or PCI	Low	H/S	oral NAC	49	2	57.64±8.4	42(85.7)	7(14.3)	600mg for 4 days started 12	136.73±100.23*	123.7±17.08** (umol/L)

										hours before contrast administration. H/S at a rate of I ml/kg/h 12 hours before and after coronary angiogram.		
				Control	51	6	56.4±6.78	42(82.4)	9(17.6)	H/S only	126.67±94.37[*]	124.4±21.89[**] (umol/L)
Kimmel et al. (2008)	CAG	Low	H/S	oral NAC	19	1	71.5±9.5	13(68.4)	6(11.6)	600 mg orally twice daily day preceding and day of procedure, total 2 days. H/S was given at 1 ml/kg/h for 24 h (12 h before and 12 h after exposure to CM.	187±88[*]	1.51±0.23[**] (mg/dL)
				Zinc (excluded)	18	2	67.2±11.4	14	4	Excluded from analysis		
				Placebo	17	1	66.8±10.4	12(70.6)	5(29.4)	H/S and matched placebo	219±105[*]	1.65±0.65[**] (mg/dL)

Hsu et al. (2007)	CAG or PCI	Low	H/S	oral NAC	11	0	44-84	7(63.6)	4(36.4)	4 doses of NAC (600 mg/twice a day, 2 doses before and 2 doses after the procedure). H/S at a rate of 1 mL/(kg • h), 12 hours before to 12 hours after the procedure.	206.5±67.5*	2.9±0.9** (mg/dL)
				Control	9	5	48-78	3(33.3)	6(66.7)	H/S only	66.7±35.8*	2.6±0.8** (mg/dL)
Carbonell et al. (2007)	CAG	Low	H/S	IV NAC	107	11	63.1±13.7	86(80.4)	21(19.6)	600 mg in 50 ml N/S over 30 min twice daily for total of 4 doses, starting at least 6 hours pre-procedure. H/S was given at least 6 h before the procedure and 12 h after CM exposure.	193±11*	0.94±0.16** (mg/dL)
				Control	109	11	60.7±11.7	79(72.5)	30(27.5)	H/S only	183±10*	0.96±0.17** (mg/dL)
Lawlor et al. (2007)	PA	NR	N/S	oral NAC	25	2	NR	19(76)	6(24)	600 mg in 30ml of ginger ale orally twice daily, day prior	158±6.1*	167±46** (umol/L)

										to and day of angioplasty. N/S was given at 1 mL/kg/h both prior to and following 12hour of the procedure.		
				Placebo	25	2	NR	17(68)	8(32)	N/S and matched placebo	163±5.8*	172±48** (umol/L)
				Control (excluded)	28	2	NR	18	10	Excluded from analysis		
Reinecke et al. (2007)	CAG	Iso	N/S	oral NAC	114	6	66.7±10.1	89(78.1)	25(21.9)	600 mg evening pre-procedure, second dose morning pre-procedure, third evening post-procedure, and the last dose was given on morning post-procedure. All patients received a hydration therapy consisting of 500 ml 5% glucose and 500 ml N/S over 12 h before, and again 500 ml 5% glucose and 500	197±80*	1.5(1.3-1.9)# (mg/dL)

										ml N/S over 12 h after CAG.		
				Haemodialysis (excluded)	113	18	67.9±9.8	89	24	Excluded from analysis		
				Control	115	7	66.7±10.6	91(79.1)	24(20.9)	Hydration therapy only	188±79*	1.4 (1.3-1.9)# (mg/dL)
Seyon et al. (2007)	CAG	Low/Iso	H/S	oral NAC	20	1	76.4±5.9	12(60)	8(40)	600 mg orally, for a total of 4 doses, with the first dose at 8:00 A.M. the day of the procedure and 3 doses post-procedure. H/S at 1 mL/kg/h 4 to 6 hours before and 12 hours after CAG.	147.5±74.75*	NR
				Control	20	2	74.7±9.7	14(70)	6(30)	H/S and matched placebo	133.68±58.04*	NR
Coyle et al. (2006)	CAG	NR	H/S	oral NAC	65	6	66.7±10	42(64.6)	23(35.4)	600 mg orally every 12 hours, 2 doses before and 2 days after administration of contrast. H/S was given at a rate of 300 mL/h for 6 hours was begun in the catheterization laboratory.	88±61*	1.16±0.38** (mg/dL)
				Control	69	1	63.3±9.2	47(68.1)	22(31.9)	H/S only	98±65*	1.10±0.44** (mg/dL)

Marenzi et al. (2006)	CAG	Low	N/S	IV NAC	115	17	62.5±13	100(87)	15(13)	Intravenous bolus of 600 mg pre-procedure and 600mg orally twice daily for 48h post-procedure, to a total dose 3000mg after intervention (total dose of 3000 mg)N/S was given at a rate of 1 ml/kg/h for 12 hours after CAG.	264±146*	Mean SCr.: 1.01(mg/dL)
				IV NAC Double Dose Group (excluded)	118	10	62.2±11	87	31	Excluded from analysis		
				Placebo	119	39	62.6±12	97(81.5)	22(18.5)	N/S and matched placebo	274±113*	Median SCr.: 1.06(mg/dL)
Moore et al. (2006)	EVAR	Low	N/S	oral NAC	11	0	71	NR	NR	600 mg orally twice daily for 2 days, starting day pre-procedure (total 3 doses pre-op) Unclear hydration protocol.	Median (IQR): 258 (210-285)	102 (76-112)# (umol/L)
				Control	9	0	72	NR	NR	N/S only	Median (IQR): 258 (200-355)	86 (81.5-99)# (umol/L)
Gomes et al. (2005)	CAG or PCI	Low	N/S	oral NAC	150	9	64.5±12	104(69.3)	46(30.7)	600 mg orally twice daily for 2	102.5±47.3*	123.76±45.0** 8 (umol/L)

										days, starting 24h pre-procedure (2 doses pre- and 2 doses post-procedure) N/S was given from 12 hours before to 12 hours after exposure to CM.		
				Placebo	151	9	64.1±12	113(74.8)	37(25.2)	N/S and matched placebo	102.8±60.4*	111.38±30.94** (umol/L)
Gulel et al. (2005)	CAG	Low	N/S	oral NAC	25	3	61.4±12.3	20(80)	5(20)	600 mg orally twice daily for 2 days, starting 24h pre-procedure	NR	1.6±0.4** mg/dL
				Control	25	2	61.5±11.6	18(72)	7(28)	IV hydration only	NR	1.8±0.6** (mg/dL)
Kotlyar et al. (2005)	CAG, PCI or PA	Low	N/S	IV NAC low dose (excluded)	20	4	66±14	15	5	Excluded from analysis		
				IV NAC	21	2	67±12	18(85.7)	3(14.3)	600mg in 100ml of 5% dextrose administered over 20 min, 1–2h before angiography and again 2–4h after angiography. N/S was commenced at 200 ml/h 2 h before the	89±32*	27.5±5.8# (umol/L)

										procedure and continued for a further 5 h after it.		
				Control	19	2	69±9	17(89.5)	2(10.5)	N/S only	86±41*	27.5±5.8# (umol/L)
Fung et al. (2004)	CAG or PCI	Low	N/S	oral NAC	46	8	68.2±8.4	34(73.9)	12(26.1)	400 mg, thrice daily the day pre- and day of contrast procedure. N/S was given at 100 mL/h from 12 hours before the procedure until 12 hours after the procedure.	135.8±66.6*	2.27±0.54** (mg/dL)
				Control	45	6	68±8.8	30(66.7)	15(33.3)	N/S only	121±66.2*	2.37±0.61** (mg/dL)
Goldenberg et al. (2004)	CAG or PCI	Low	H/S	oral NAC	41	4	71±9	35(85.4)	6(14.6)	600 mg orally thrice daily for 2 days, starting 24h pre-procedure. H/S was given at a rate of 1 ml/kg/h for 12 h before and 12 h after administration of CM.	111±43*	2±0.4** (mg/dL)
				Placebo	39	3	69±10	31(79.5)	8(20.5)	H/S and matched placebo	121±49*	1.9±0.3* (mg/dL)

Miner et al. (2004)	CAG or PCI	Iso	H/S	oral NAC	95	9	71±8	65(68.4)	30(31.6)	6000mg or 4000mg oral NAC. H/S was given at 75 mL/hour for at least 24 hours beginning at the time of enrollment.	344±211*	124±49* (umol/L)
				Control	85	19	69±11	56(65.9)	29(34.1)	H/S only	350±187*	130±58** (umol/L)
Ochoa et al. (2004)	CAG or PCI	Low/Iso	N/S	oral NAC	36	3	73±8	16(44.4)	20(55.6)	1000 mg diluted in 20 mL of diet cola administered orally 1h pre- and 4h post-procedure. N/S 150 mL/h beginning 4 hours before the procedure and continuing for 6 hours after the procedure.	NR	2.02±0.56** (mg/dL)
				Placebo	44	11	70±12	18(40.9)	26(59.1	N/S and matched placebo	NR	1.93±0.53** (mg/dL)
Rashid et al. (2004)	CAG, PCI or PA	Low	N/S	IV NAC	46	3	72.09±12.34	27(58.7)	16(41.3)	1g per bag of N/S. N/S (500 mL over 4 to 6 hours) 6 to 12 hours prior to the procedure	135.4±62.7*	109.9±41.15** (umol/L)

										and again after the procedure.		
				Control	48	3	67.75±12.32	33(84.6)	15(15.4)	N/S only	151.2±75.6*	124.3±63.47** (umol/L)
Webb et al. (2004)	CAG or PCI	Low	N/S	IV NAC	242	25	70.8±10.3	144(59.5)	98(40.5)	500 mg immediately pre-procedure. All patients received 200 mL of N/S before the procedure, followed by 1.5 mL/kg/h for 6 hours or until discharge.	Median (IQR): 120 (80-186)	141 (125-166)# (umol/L)
				Placebo	245	24	70.0±9.4	152(62)	93(38)	N/S and matched placebo	Median (IQR): 120 (80-155)	142 (124-167)# (umol/L)
Baker et al. (2003)	CAG or PCI	Iso	N/S	IV NAC	41	2	67.4±10.3	37(90.2)	4(9.8)	150 mg/kg in 500 ml saline (0.9%) over 30 min immediately before contrast exposure followed by 50 mg/kg in 500 ml N/S over the subsequent 4 hours	238±155*	1.85±0.59** (mg/dL)
				Control	39	8	70.9±8.8	33(84.6)	6(15.4)	N/S at 1 ml/kg/h for 12 h pre- and post-procedure.	222±162*	1.75±0.41** (mg/dL)
Efrati et al. (2003)	CAG	Low	H/S	oral NAC	24	0	68±1.9	21(87.5)	3(12.5)	1 g orally twice daily 24 hours pre- and 24 hours post-CAG	142±25.3*	135.25±6.19** (umol/L)

									H/S at a rate of 1 mL/kg/h for 12 hours before and 12 hours after CAG.		
				Placebo	25	2	66±1.9	23(92)	2(8)	H/S and matched placebo	138±33.7*	131.7±6.19** (umol/L)
Kay et al. (2003)	CAG or PCI	Low	N/S	oral NAC	102	4	69	61(59.8)	41(40.2)	600 mg orally twice daily at the day preceding and day of the procedure. N/S was given at a rate of 1 mL/kg/h for 12 hours before and for 6 hours after CM exposure.	Mean (IQR): 130 (75-320)	#1.24 (0.77-2.99) (mg/dL)
				Placebo	98	12	69	62(63.3)	36(36.7)	N/S and matched placebo	Mean (IQR): 120 (70-380)	1.26 (0.75-3.64)# (mg/dL)
Kefer et al. (2003)	CAG or PCI	Low	N/S	IV NAC	53	2	61±10	41(77.4)	12(22.6)	1200mg in 200ml N/S in two separate 60 minute infusions, first 12hr pre-procedure, and second following administration of contrast.	NR	NR
				Control	51	3	64±10	39(76.5)	12(23.5)	N/S and matched placebo	NR	NR

MacNeill et al. (2003)	CAG or PCI	Low	H/S	oral NAC	21	1	72.1±8.8	16(76.2)	5(23.8)	600mg twice daily commenced day of procedure, for total of 5 doses. H/S at a rate of 1 ml/kg/h for 12 h for in-patients and 2 ml/kg/h for 4 h for day-case patients. Postprocedural hydration with H/S at 75 ml/h for 12 hours.	103±52*	1.89±0.38** (mg/dL)
				Control	22	7	72.9±10.3	21(95.5)	1(4.5)	H/S and matched placebo	116±63.3*	1.88±0.41** (mg/dL)
Oldemeyer et al. (2003)	CAG	Low	H/S	oral NAC	49	8	77±9	27(55.1)	22(44.9)	1500 mg orally in 120 mL of carbonated beverage commenced evening pre-procedure (total 4 doses). H/S at 1 mL/kg/h for 12 hours before and 12 hours after CAG.	134±71*	NR
				Placebo	47	3	75±8	26(55.3)	21(44.7)	H/S with matched placebo	127±73*	NR
	PCI	Low	H/S	oral NAC	45	8	71±10	28(62.2)	17(37.8)	600 mg orally twice daily	1.52±0.81* (ml/kg)	**2.20±0.73** (mg/dL)

Allaqaband et al. (2002)										starting the day before PCI. H/S at the rate of 1 ml/kg/h for 12 h prior to PCI, during the procedure, and for 12 h after PCI.		
				Fenoldopam (excluded)	38	6	71±10	19	19	Excluded from analysis		
				Control	40	8	71±10	24(60)	16(40)	H/S only	1.47±0.90* (ml/kg)	2.03±0.79** (mg/dL)
Briguori et al. (2002)	CAG, PCI or PA	Low	H/S	oral NAC	92	6	64±9	77(83.7)	15(16.3)	600 mg orally every 12 hours before and on day of administration, total of 2 days. H/S at a rate of 1 ml/kg/h for 12 h before and 12 h after administration of CM.	194±127*	1.52±0.43** (mg/dL)
				Control	91	10	64±9	81(89)	10(11)	H/S only	200±144*	1.54±0.36** (mg/dL)
Durham et al. (2002)	CAG or PCI	Low	H/S	oral NAC	38	10	69.8±9.7	24(63.2)	14(36.8)	1200 mg orally 1 hour pre- and 3 hours post-CM. H/S at a rate of 1 ml/kg/h for 12 h before and 12 h after	77.4±35.9*	2.2±0.4** (mg/dL)

										administration of CM.		
				Placebo	41	9	71.4±12.2	28(68.3)	13(31.7	H/S with matched placebo	84.7+42.1*	2.3±0.5** (mg/dL)
Diaz-Sandoval et al. (2002)	CAG or PCI	Low	H/S	oral NAC	25	2	74±2	17(68)	8(32)	NAC 600mg orally, twice daily for 4 doses. H/S at 1 ml/kg/h for 2 to 12 hours before and for 12 hours after the procedure.	NR	1.66±0.06** (mg/dL)
				Placebo	29	13	72±2	26(89.7)	3(10.3)	H/S with matched placebo	NR	1.56±0.05** (mg/dL)
Shyu et al. (2002)	CAG	Low	H/S	oral NAC	60	2	70±7	42(70)	18(30)	400 mg twice daily orally, on the day pre- and day of procedure, for a total of two days. H/S at a rate of 1 ml/kg/h for 12 h before and 12 h after CAG.	119±3*	2.8±0.8** (mg/dL)
				Placebo	61	15	70±7	40(65.6)	21(34.4)	H/S with matched placebo	115±48*	2.8±0.8** (mg/dL)

Abbreviations: CA-AKI, Contrast-associated acute kidney injury; CAG, Coronary angiography; ClCr, Creatinine clearance by Cockcroft-Gault equation; CM, contrast media; eGFR, Estimated glomerular filtration rate; EVAR, Endovascular aneurysm repair; H, Hydration; H/S, Half saline; IPC: Ischemic preconditioning; IQR, interquartile range; IV, Intravenous; NAC, N-acetylcysteine; N/S, Normal saline; NR, not reported; PA, Peripheral angioplasty; PCI, Percutaneous intervention; PTX: Pentoxifylline; RIPC, Remote ischemic preconditioning; SD, Standard deviation; SCr., Serum creatinine; OSM, Osmolarity

* Mean ± SD

[**] Mean Serum Creatinine (± SD)

[#] Median Serum Creatinine (IQR)

Table S2. The number of each intervention within network

Intervention	Number
H	5738
H+oral NAC	5603
H+IV NAC	1571
H+oral NAC+statin	954
H+oral NAC+NaHCO3	388
H+IV NAC+oral NAC	244
H+oral NAC+RIPC	211
H+IV NAC+RIPC	111
H+oral NAC+sham RIPC	100
H+oral NAC+Febuxostat	60
H+oral NAC+Pentoxifylline	55
H+oral NAC+NaHCO3+Pentoxifylline	40

Abbreviations: H, Hydration; NAC, N-acetylcysteine; RIPC, Remote ischemic preconditioning

Figure S1. Risk of bias assessment

	D1	D2	D3	D4	D5	Overall
Sarhan, 2023	+		+	+	+	
R. Sahu, 2021	+	+	+	+	+	+
K. Samadi, 2020				+	+	x
K. Stokfisz, 2020	+	+	+	+	+	+
Aslanabadi, 2019	+	+	+	+	+	+
F. Barzi, 2019	+	+	+	+	+	+
Ghaemian, 2018	+	+	+	+	+	+
Wojciechowska, 2018	+	+	+	+	+	+
Valappil, 2018	+	+	+	+	+	+
B. Biernacka-Fialkowska, 2018	+	+	+	+	+	+
Saied Shalaby, 2018	+	+	+	+	+	+
Sadineni, 2017	+	+	+	+	+	+
M. H. Syed, 2017	+	+	+	+	+	+
Khosravi, 2016	+	+	+	+	+	+
A. Pezeshgi, 2015	+	+	+	+	+	+
Erturk, 2014	+	+	+	+	+	+
Inda-Filho, 2014	+	+	+	+	+	+
Kumar, 2014	+			+	+	x
Leoncini, 2014	+	+	+	+	+	+
Yang, 2014	+		+	+	+	
K. Mahmoodi, 2014	+		+	+	+	
P. Thayssen, 2014	+	+	+	+	+	+
Albabtain, 2013	+	+	+	+	+	+
Berwanger, 2013	+	+	+	+	+	+
Brueck, 2013	+	+	+	+	+	+
Aslanger, 2012	+	+	+	+	+	+
Er F. 2012	+		+	+	+	
Gunebakmaz, 2012	+		+	+	+	
Jaffery, 2012	+		+	+	+	
Koc, 2012	+		+	+	+	
A.C.T Investigators, 2011	+	+	+	+	+	+
Sadat, 2011	+		+	+	+	
Saitoh, 2011	+	+	+	+	+	+
Carbonell, 2010	+	+	+	+	+	+
Kinbara, 2010	+	+	+	+	+	+
Ozhan, 2010	+		+	+	+	
Thiele, 2010	+	+	+	+	+	+
Toso, 2010	+	+	+	+	+	+
Amini, 2009	+	+	+	+	+	+
Baskurt, 2009			+	+	+	
Ferrario, 2009	+	+	+	+	+	+
J. A. Ratcliffe, 2009	+	+		+	+	+
Izani, 2008	+	+	+	+	+	+
Kimmel, 2008	+	+	+	+	+	+
Carbonell, 2007	+	+	+	+	+	+
Lawlor, 2007	+	+	+	+	+	+
Reinecke, 2007	+	+	+	+	+	+
Seyon, 2007	+	+	+	+	+	+
C. H. Hsu, 2007	+	+	+	+	+	+
Coyle, 2006	+	+	+	+	+	+
Marenzi, 2006	+	+	+	+	+	+
Moore, 2006		+	+	+	+	
Gomes, 2005	x	+	+	+	+	x
Gulel, 2005	+		+	+	+	
Kotlyar, 2005	+	+		+	+	
Fung, 2004	+		+	+	+	
Goldenberg, 2004	+	+	+	+	+	+
Miner, 2004	+			+	+	
Ochoa, 2004	+	+	+	+	+	+
Rashid, 2004	+	+	+	+	+	+
Webb, 2004	+	+	+	+	+	+
Baker, 2003	+	+	+	+	+	+
Efrati, 2003	+	+	+	+	+	+
Kay, 2003	+	+	+	+	+	+
Kefer, 2003	+	+	+	+	+	+
MacNeill, 2003	+	+	+	+	+	+
Oldemeyer, 2003	+	+		+	+	+
Allaqaband, 2002	+	+	+	+	+	+
Briguori, 2002	+		+	+	+	+
Durham, 2002	+	+	+	+	+	+
Shyu, 2002	+	+		+	+	
L. J. Diaz-Sandoval, 2002	+	+		+	+	

The overall figure(left) and the statistical proportions for each risk(below) were presented, using Cochrane's risk of bias assessment tool (Rob 2.0). Studies were categorized as being at high, some concerns, or low risk of bias depending on the presence of selection bias, performance bias, detection bias, attrition bias, or reporting bias. Green represents "low risk of bias"; yellow "some concerns"; red, "high risk of bias".

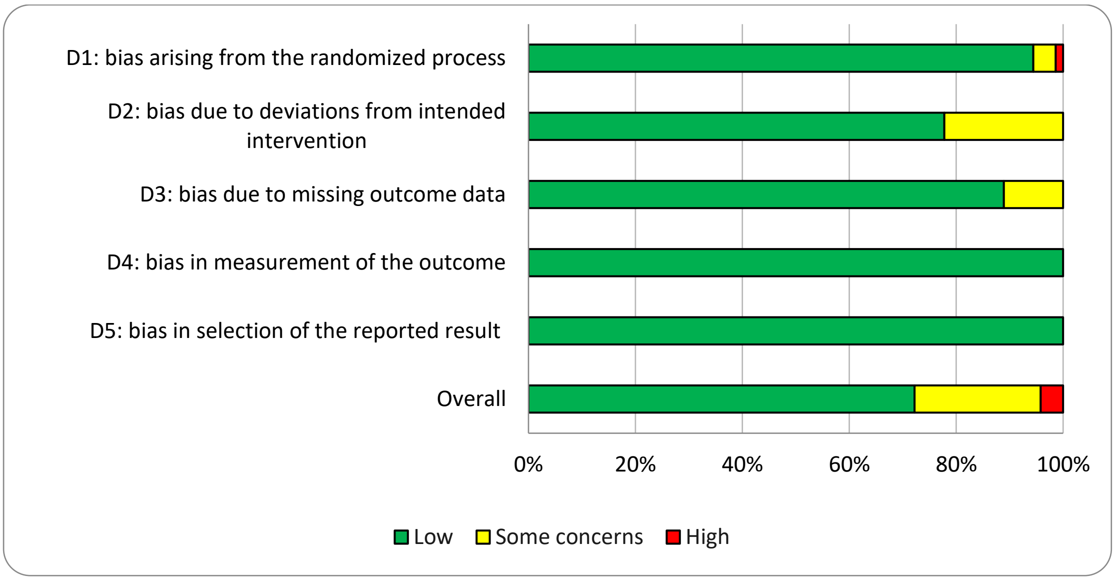

Figure S2. Funnel plot for the assessment of publication bias of the included studies in this network meta-analysis.

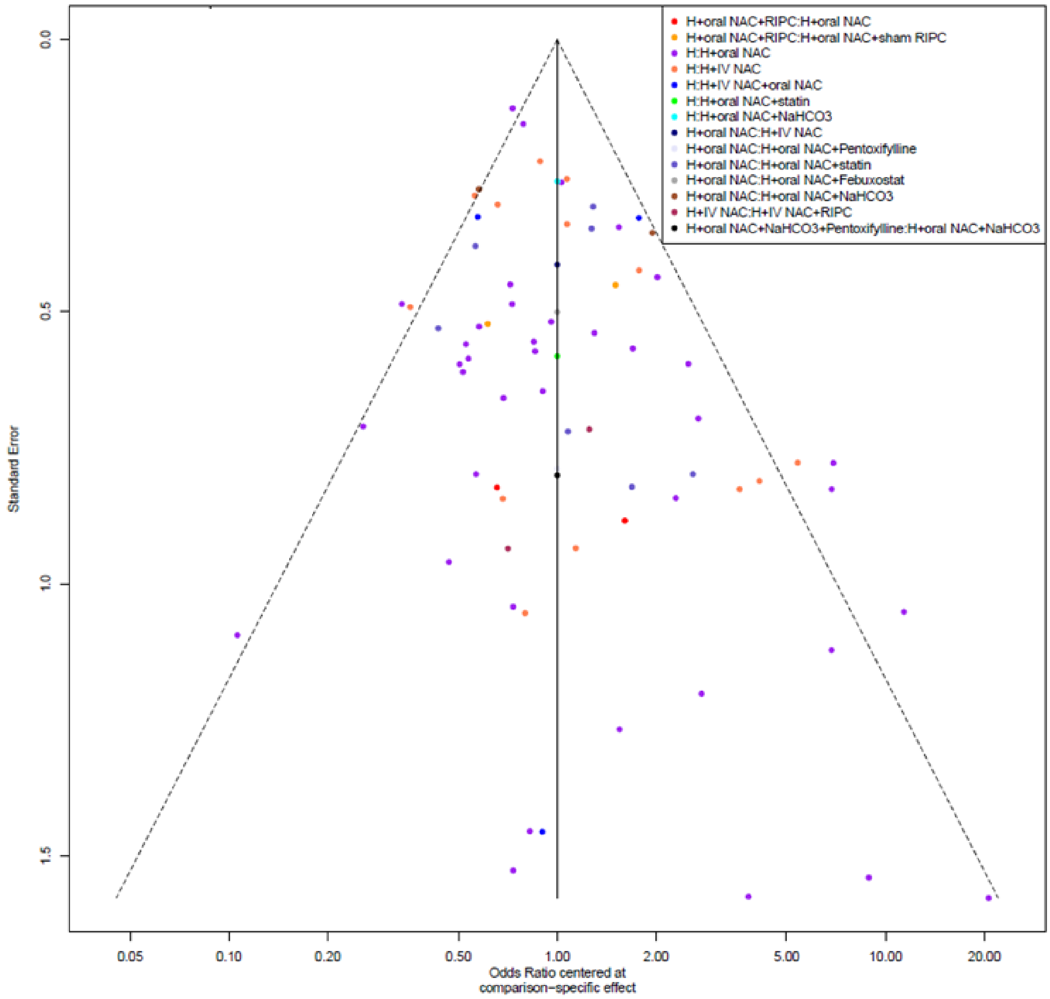

Abbreviations: H, Hydration; NAC, N-acetylcysteine**;** RIPC, Remote ischemic preconditioning

Figure S3. Forest plot for different strategies to prevent the risk of CA-AKI among different subgroup

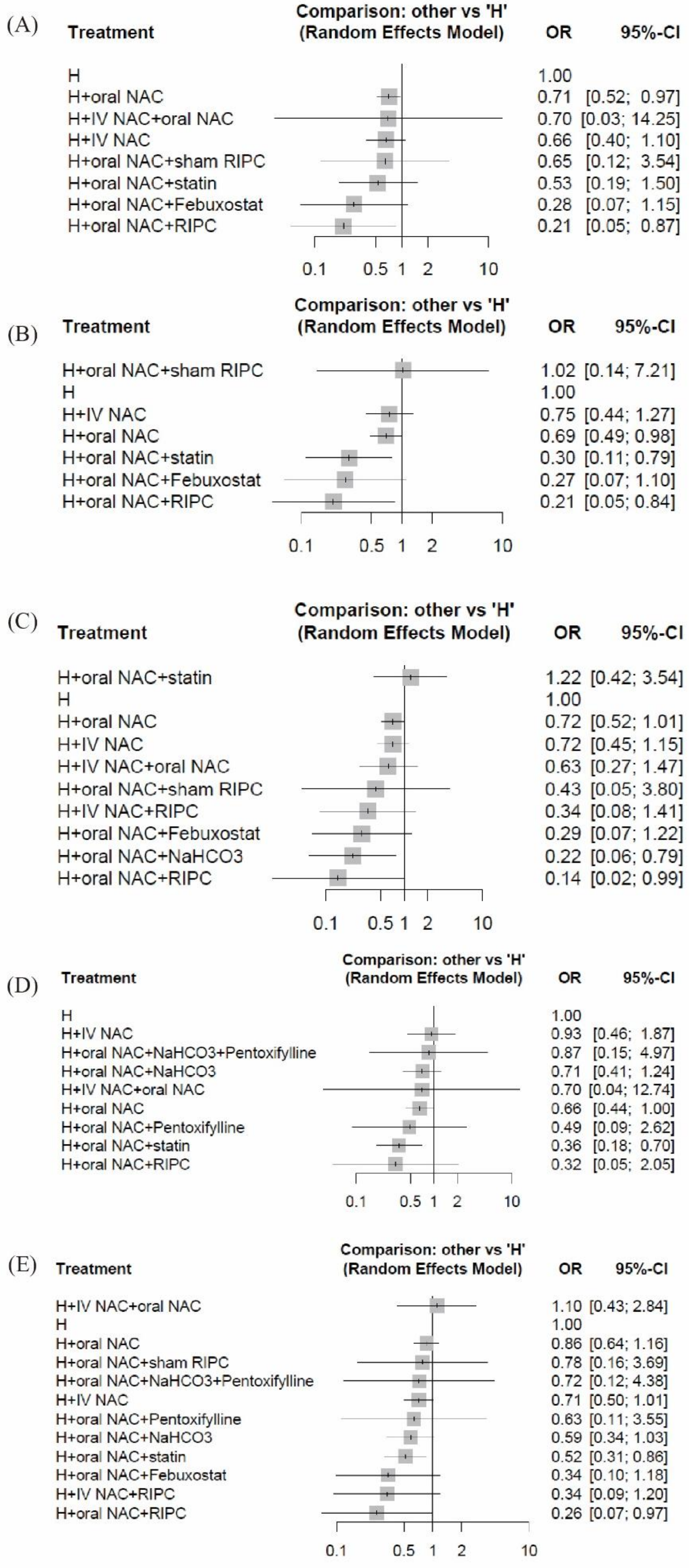

A. **Figure S3A. Forest plot for different strategies to prevent the risk of CA-AKI among CKD(1)# patients. (45 out of 72 trials)**
   #CKD(1) was defined as an eGFR(or ClCr) of 60 mL/min/1.73 m^2 or less, or a Creatinine level of 1.2 mg/dL or higher.
B. **Figure S3B. Forest plot for different strategies to prevent the risk of CA-AKI among CKD(2)# patients. (34 out of 72 trials)**
   #CKD(2) was defined as an eGFR(or ClCr) of 45 mL/min/1.73 m^2 or less, or a Creatinine level of 1.45 mg/dL or higher.
C. **Figure S3C. Forest plot for different strategies to prevent the risk of CA-AKI among the patients with low-osmolar contrast# (48 out of 72 trials)**
   #Information on the type of contrast was obtained from each article. The articles included different type of contrast were excluded.
D. **Figure S3D. Forest plot for different strategies to prevent the risk of CA-AKI among the patients with iso-osmolar contrast¥ (18 out of 72 trials)**
   ¥Information on the type of contrast was obtained from each article. The articles included different type of contrast were excluded.
E. **Figure S3E. Forest plot for different strategies to prevent the risk of CA-AKI with the studies conducted after 2010 (44 out of 72 trials)**

Abbreviations: CA-AKI, Contrast-associated acute kidney injury; CI, Confidence interval; ClCr, Creatinine clearance by Cockcroft-Gault equation; H, Hydration; NAC, N-acetylcysteine**;** RIPC, Remote ischemic preconditioning; OR, Odds ratio

Table S3. P-score rankings of different interventions for preventing CA-AKI

The P-score represents the likelihood of an intervention having a greater effect compared to other treatments, with a P-score of one indicating the highest probability.

Intervention	P-score
H+oral NAC+RIPC	0.8537
H+oral NAC+Febuxostat	0.7577
H+IV NAC+RIPC	0.7214
H+oral NAC+statin	0.6294
H+oral NAC+NaHCO3	0.5388
H+oral NAC+Pentoxifylline	0.4857
H+IV NAC+oral NAC	0.4423
H+oral NAC+NaHCO3+Pentoxifylline	0.4253
H+oral NAC+sham RIPC	0.3868
H+IV NAC	0.3598
H+oral NAC	0.2917
H	0.1073

Abbreviations: CA-AKI, Contrast-associated acute kidney injury; H, Hydration; NAC, N-acetylcysteine; RIPC, Remote ischemic preconditioning

Figure S4. Forest plot displaying the separation of direct and indirect evidence for interventions aimed at preventing CA-AKI, and assessing the consistency between each comparison within our network meta-analysis.

Comparison	Number of Studies	Direct Evidence	I2	OR	95%-CI
**H+IV NAC:H**					
Direct estimate	13	0.92	46.5%	0.71	[0.51; 1.00]
Indirect estimate				0.71	[0.23; 2.24]
Network estimate				0.71	[0.52; 0.99]
**H+oral NAC:H**					
Direct estimate	39	0.91	41.2%	0.73	[0.58; 0.93]
Indirect estimate				1.32	[0.64; 2.72]
Network estimate				0.78	[0.62; 0.97]
**H+oral NAC+NaHCO3:H**					
Direct estimate	1	0.38		0.74	[0.29; 1.87]
Indirect estimate				0.45	[0.22; 0.95]
Network estimate				0.55	[0.31; 0.97]
**H+oral NAC+statin:H**					
Direct estimate	1	0.13		1.76	[0.44; 7.00]
Indirect estimate				0.39	[0.23; 0.66]
Network estimate				0.47	[0.29; 0.77]
**H+IV NAC:H+oral NAC**					
Direct estimate	1	0.12		0.92	[0.30; 2.83]
Indirect estimate				0.92	[0.61; 1.39]
Network estimate				0.92	[0.63; 1.35]
**H+oral NAC:H+oral NAC+NaHCO3**					
Direct estimate	2	0.65	86.4%	1.68	[0.83; 3.39]
Indirect estimate				1.04	[0.40; 2.71]
Network estimate				1.42	[0.81; 2.50]
**H+oral NAC:H+oral NAC+statin**					
Direct estimate	7	0.90	22.7%	1.91	[1.19; 3.07]
Indirect estimate				0.43	[0.11; 1.73]
Network estimate				1.63	[1.04; 2.56]

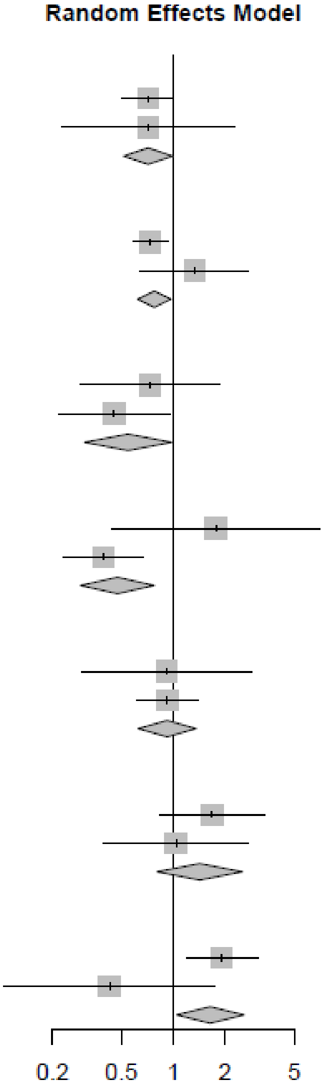

Abbreviations: CA-AKI, Contrast-associated acute kidney injury; H, Hydration; NAC, N-acetylcysteine; RIPC, Remote ischemic preconditioning

Table S4. Consistency test by side-splitting model

comparison	TE	seTE	lower	upper	statistic	p
H+IV NAC:H	-0.3367	0.166529	-0.66309	-0.01031	-2.02186	0.043191
H+IV NAC+oral NAC:H	-0.46104	0.354051	-1.15496	0.232892	-1.30217	0.192857
H+IV NAC+RIPC:H	-1.08792	0.659458	-2.38044	0.204589	-1.64973	0.098999
H+oral NAC:H	-0.25416	0.112624	-0.47489	-0.03342	-2.25667	0.024029
H+oral NAC+Febuxostat:H	-1.17992	0.649611	-2.45314	0.09329	-1.81636	0.069316
H+oral NAC+NaHCO3:H	-0.60423	0.29518	-1.18277	-0.02569	-2.04698	0.04066
H+oral NAC+NaHCO3+Pentoxifylline:H	-0.40356	0.941935	-2.24972	1.442601	-0.42843	0.668334
H+oral NAC+Pentoxifylline:H	-0.56126	0.890816	-2.30722	1.184711	-0.63005	0.528664
H+oral NAC+RIPC:H	-1.45342	0.674806	-2.77602	-0.13083	-2.15383	0.031253
H+oral NAC+sham RIPC:H	-0.35398	0.807599	-1.93685	1.228881	-0.43832	0.661157
H+oral NAC+statin:H	-0.74575	0.250249	-1.23623	-0.25527	-2.98003	0.002882
H+IV NAC:H+IV NAC+oral NAC	0.124337	0.39126	-0.64252	0.891192	0.317788	0.750646
H+IV NAC:H+IV NAC+RIPC	0.751226	0.638085	-0.4994	2.00185	1.177314	0.23907
H+IV NAC:H+oral NAC	-0.08254	0.195675	-0.46606	0.300973	-0.42184	0.673143
H+IV NAC:H+oral NAC+Febuxostat	0.843226	0.669029	-0.46805	2.154498	1.260374	0.207534
H+IV NAC:H+oral NAC+NaHCO3	0.26753	0.336906	-0.39279	0.927855	0.794079	0.427149
H+IV NAC:H+oral NAC+NaHCO3+Pentoxifylline	0.06686	0.955833	-1.80654	1.940258	0.069949	0.944234
H+IV NAC:H+oral NAC+Pentoxifylline	0.224557	0.905073	-1.54935	1.998467	0.248109	0.80405
H+IV NAC:H+oral NAC+RIPC	1.116723	0.693519	-0.24255	2.475994	1.610227	0.107348
H+IV NAC:H+oral NAC+sham RIPC	0.017285	0.823299	-1.59635	1.630921	0.020995	0.983249
H+IV NAC:H+oral NAC+statin	0.409052	0.297412	-0.17386	0.991969	1.375374	0.169016
H+IV NAC+oral NAC:H+IV NAC+RIPC	0.626889	0.74849	-0.84012	2.093901	0.837538	0.40229

H+IV NAC+oral NAC:H+oral NAC	-0.20688	0.371532	-0.93507	0.521309	-0.55683	0.577643
H+IV NAC+oral NAC:H+oral NAC+Febuxostat	0.718889	0.739829	-0.73115	2.168927	0.971696	0.331202
H+IV NAC+oral NAC:H+oral NAC+NaHCO3	0.143193	0.460959	-0.76027	1.046657	0.310641	0.756074
H+IV NAC+oral NAC:H+oral NAC+NaHCO3+Pentoxifylline	-0.05748	1.006277	-2.02975	1.91479	-0.05712	0.95445
H+IV NAC+oral NAC:H+oral NAC+Pentoxifylline	0.100219	0.958595	-1.77859	1.979031	0.104548	0.916734
H+IV NAC+oral NAC:H+oral NAC+RIPC	0.992385	0.762047	-0.5012	2.48597	1.302262	0.192827
H+IV NAC+oral NAC:H+oral NAC+sham RIPC	-0.10705	0.881798	-1.83534	1.621241	-0.1214	0.903373
H+IV NAC+oral NAC:H+oral NAC+statin	0.284715	0.433563	-0.56505	1.134483	0.656687	0.511382
H+IV NAC+RIPC:H+oral NAC	-0.83377	0.667414	-2.14188	0.474338	-1.24925	0.211572
H+IV NAC+RIPC:H+oral NAC+Febuxostat	0.092	0.924528	-1.72004	1.904041	0.09951	0.920733
H+IV NAC+RIPC:H+oral NAC+NaHCO3	-0.4837	0.721567	-1.89794	0.930549	-0.67034	0.50264
H+IV NAC+RIPC:H+oral NAC+NaHCO3+Pentoxifylline	-0.68437	1.149247	-2.93685	1.568116	-0.59549	0.551515
H+IV NAC+RIPC:H+oral NAC+Pentoxifylline	-0.52667	1.107389	-2.69711	1.643773	-0.4756	0.634362
H+IV NAC+RIPC:H+oral NAC+RIPC	0.365496	0.942401	-1.48158	2.212569	0.387835	0.698138
H+IV NAC+RIPC:H+oral NAC+sham RIPC	-0.73394	1.041621	-2.77548	1.307598	-0.70461	0.48105
H+IV NAC+RIPC:H+oral NAC+statin	-0.34217	0.703993	-1.72198	1.037627	-0.48605	0.626934

H+oral NAC:H+oral NAC+Febuxostat	0.925769	0.639774	-0.32816	2.179703	1.447027	0.147889
H+oral NAC:H+oral NAC+NaHCO3	0.350074	0.289188	-0.21672	0.916872	1.210539	0.226072
H+oral NAC:H+oral NAC+NaHCO3+Pentoxifylline	0.149403	0.940075	-1.69311	1.991915	0.158927	0.873727
H+oral NAC:H+oral NAC+Pentoxifylline	0.3071	0.883668	-1.42486	2.039057	0.347529	0.728194
H+oral NAC:H+oral NAC+RIPC	1.199266	0.665341	-0.10478	2.503311	1.802482	0.07147
H+oral NAC:H+oral NAC+sham RIPC	0.099829	0.799707	-1.46757	1.667226	0.124832	0.900657
H+oral NAC:H+oral NAC+statin	0.491596	0.229387	0.042006	0.941185	2.143088	0.032106
H+oral NAC+Febuxostat:H+oral NAC+NaHCO3	-0.5757	0.702097	-1.95178	0.800389	-0.81997	0.412235
H+oral NAC+Febuxostat:H+oral NAC+NaHCO3+Pentoxifylline	-0.77637	1.137124	-3.00509	1.452355	-0.68275	0.494767
H+oral NAC+Febuxostat:H+oral NAC+Pentoxifylline	-0.61867	1.090953	-2.7569	1.51956	-0.56709	0.570653
H+oral NAC+Febuxostat:H+oral NAC+RIPC	0.273496	0.923033	-1.53561	2.082608	0.296302	0.767
H+oral NAC+Febuxostat:H+oral NAC+sham RIPC	-0.82594	1.02413	-2.8332	1.181317	-0.80648	0.419966
H+oral NAC+Febuxostat:H+oral NAC+statin	-0.43417	0.679653	-1.76627	0.897922	-0.63882	0.522942
H+oral NAC+NaHCO3:H+oral NAC+NaHCO3+Pentoxifylline	-0.20067	0.894489	-1.95384	1.552495	-0.22434	0.822492
H+oral NAC+NaHCO3:H+oral NAC+Pentoxifylline	-0.04297	0.929784	-1.86532	1.77937	-0.04622	0.963136
H+oral NAC+NaHCO3:H+oral NAC+RIPC	0.849192	0.725472	-0.57271	2.271091	1.170538	0.241784
H+oral NAC+NaHCO3:H+oral NAC+sham RIPC	-0.25024	0.850389	-1.91698	1.416487	-0.29427	0.768551

H+oral NAC+NaHCO3:H+oral NAC+statin	0.141522	0.367803	-0.57936	0.862402	0.384777	0.700403
H+oral NAC+NaHCO3+Pentoxifylline:H+oral NAC+Pentoxifylline	0.157697	1.290197	-2.37104	2.686437	0.122227	0.902719
H+oral NAC+NaHCO3+Pentoxifylline:H+oral NAC+RIPC	1.049863	1.151703	-1.20743	3.307159	0.911574	0.361993
H+oral NAC+NaHCO3+Pentoxifylline:H+oral NAC+sham RIPC	-0.04957	1.234209	-2.46858	2.369431	-0.04017	0.96796
H+oral NAC+NaHCO3+Pentoxifylline:H+oral NAC+statin	0.342193	0.967155	-1.5534	2.237782	0.353814	0.723479
H+oral NAC+Pentoxifylline:H+oral NAC+RIPC	0.892166	1.106141	-1.27583	3.060162	0.806557	0.419922
H+oral NAC+Pentoxifylline:H+oral NAC+sham RIPC	-0.20727	1.191805	-2.54317	2.128624	-0.17391	0.861933
H+oral NAC+Pentoxifylline:H+oral NAC+statin	0.184496	0.912955	-1.60486	1.973854	0.202086	0.839849
H+oral NAC+RIPC:H+oral NAC+sham RIPC	-1.09944	0.443681	-1.96904	-0.22984	-2.47799	0.013212
H+oral NAC+RIPC:H+oral NAC+statin	-0.70767	0.703774	-2.08704	0.671701	-1.00554	0.314639
H+oral NAC+sham RIPC:H+oral NAC+statin	0.391767	0.831956	-1.23884	2.02237	0.470899	0.637713

Abbreviations: H, Hydration; NAC, N-acetylcysteine**;** RIPC, Remote ischemic preconditioning

Figure S5. Scatter plot: distribution of treatment absolute effects on CA-AKI across different interventions

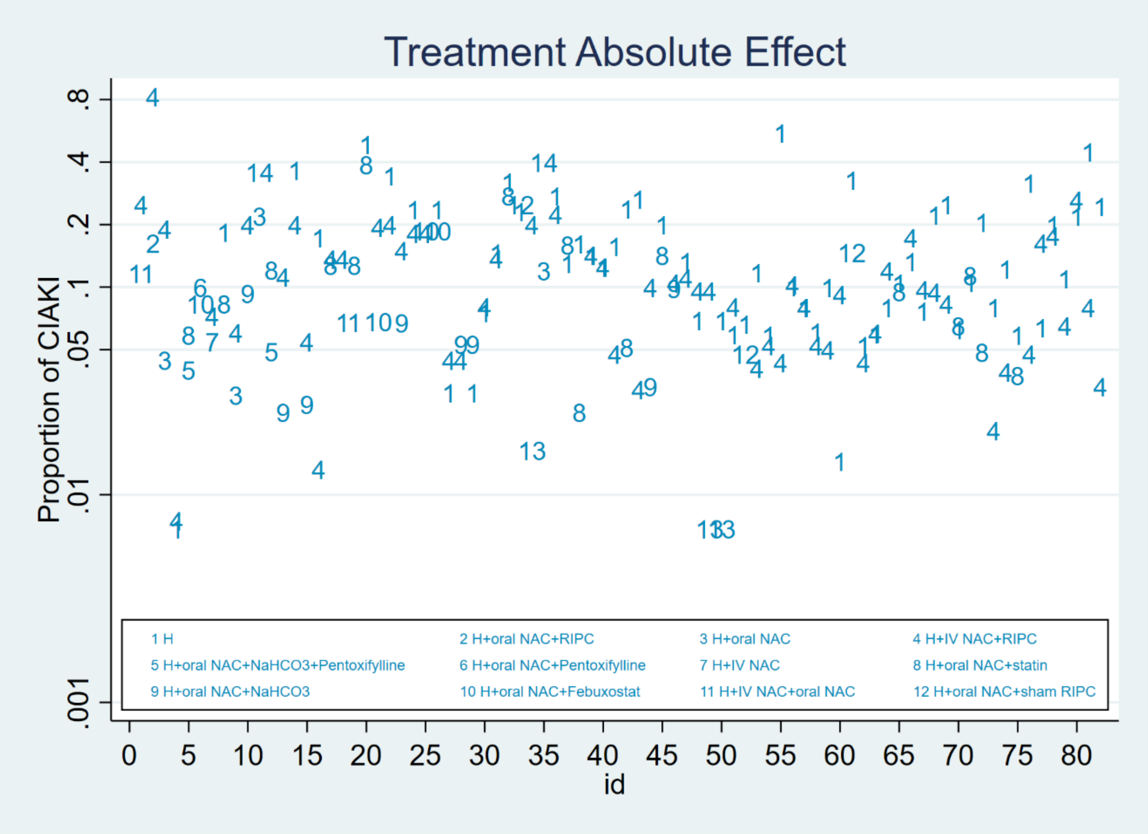

Abbreviations: CA-AKI, Contrast-associated acute kidney injury; H, Hydration; NAC, N-acetylcysteine; RIPC, Remote ischemic preconditioning

Table S5. GRADE in CA-AKI events across different intervention comparisons

comparison	Preliminary rating (No imprecision domain)			Incoherence	Imprecision	Final GRADE
	Direct outcome	Indirect outcome	Direct estimate outcome			
Hydration vs. Hydration + IV NAC	No rate down	No rate down	No rate down	No concerns	No concerns	High
Hydration vs. Hydration + oral NAC	No rate down	No rate down	No rate down	Major concerns[a]	No concerns	Low
Hydration vs. Hydration + oral NAC+ NaHCO3	No rate down	No rate down	No rate down	No concerns	Major concerns[b]	Low
Hydration vs. Hydration + oral NAC + statin	No rate down	No rate down	No rate down	Major concerns[a]	Major concerns[b]	Very low
Hydration vs. Hydration + oral NAC + IV NAC	No rate down	No rate down	No rate down	No concerns	Major concerns[b]	Low
Hydration + oral NAC vs. Hydration + oral NAC+ NaHCO3	No rate down	No rate down	No rate down	No concerns	Major concerns[b]	Low
Hydration + oral NAC vs. Hydration + oral NAC+ statin	No rate down	No rate down	No rate down	Major concerns[a]	No concerns	Low

[a] There is no coherence between the direct outcome and the indirect outcome.

[b] There is no precise outcome in network results when using minimal contextualized assessment.
